# Personal financial incentives for changing habitual health-related behaviors: A systematic review and meta-analysis

**DOI:** 10.1016/j.ypmed.2015.03.001

**Published:** 2015-06

**Authors:** Eleni Mantzari, Florian Vogt, Ian Shemilt, Yinghui Wei, Julian P.T. Higgins, Theresa M. Marteau

**Affiliations:** a Health Psychology Section, King's College London, London, UK; b Institute of Pharmaceutical Science, King's College London, London, UK; c Behaviour and Health Research Unit, University of Cambridge, Cambridge, UK; d MRC Clinical Trials Unit Hub for Trials Methodology Research, MRC Clinical Trials Unit, London, UK; e School of Social and Community Medicine, University of Bristol, Bristol UK; f Centre for Reviews and Dissemination, University of York, York, UK

**Keywords:** Financial incentives, Health-related behavior, Systematic review, Meta-analysis, Health promotion

## Abstract

**Objectives:**

Uncertainty remains about whether personal financial incentives could achieve sustained changes in health-related behaviors that would reduce the fast-growing global non-communicable disease burden. This review aims to estimate whether: i. financial incentives achieve sustained changes in smoking, eating, alcohol consumption and physical activity; ii. effectiveness is modified by (a) the target behavior, (b) incentive value and attainment certainty, (c) recipients' deprivation level.

**Methods:**

Multiple sources were searched for trials offering adults financial incentives and assessing outcomes relating to pre-specified behaviors at a minimum of six months from baseline. Analyses included random-effects meta-analyses and meta-regressions grouped by timed endpoints.

**Results:**

Of 24,265 unique identified articles, 34 were included in the analysis. Financial incentives increased behavior-change, with effects sustained until 18 months from baseline (OR: 1.53, 95% CI 1.05–2.23) and three months post-incentive removal (OR: 2.11, 95% CI 1.21–3.67). High deprivation increased incentive effects (OR: 2.17; 95% CI 1.22–3.85), but only at > 6–12 months from baseline. Other assessed variables did not independently modify effects at any time-point.

**Conclusions:**

Personal financial incentives can change habitual health-related behaviors and help reduce health inequalities. However, their role in reducing disease burden is potentially limited given current evidence that effects dissipate beyond three months post-incentive removal.

## Highlights

• Sustained changes in health behaviours would reduce non-communicable disease burden.
• Personal financial incentives can change habitual health-behaviours.
• Personal financial incentives may help reduce health inequalities.
• The impact of financial incentives is not sustained long after incentive removal.
• The role of financial incentives in reducing disease burden is potentially limited.

## Introduction

Smoking, poor diet-related behaviors, excessive alcohol consumption, and physical inactivity contribute to the development of major non-communicable diseases, i.e. cardiovascular diseases, type 2 diabetes, cancer and chronic respiratory diseases (Andersen et al., 2000, Batty et al., 2008, Batty et al., 2001, Cox et al., 2000, He et al., 2007, Heidemann et al., 2008, Teo et al., 2006), which together account for more than 50% of preventable premature deaths worldwide (3four50.com, 2011, WHO, 2012). The World Health Assembly has recently pledged to reduce non-communicable diseases by 25% by 2025 (WHO, 2013). Offering individuals personal financial incentives to change their health-related behavior could contribute to attaining this ambitious target, but uncertainty remains about the effectiveness of such schemes.

Personal financial incentives have been shown to be effective in changing several non-habitual health-related behaviors, including undergoing vaccinations, attending screening, and adhering to healthcare treatments (Jochelson, 2007, Kane et al., 2004, Sutherland et al., 2008). Whilst evidence indicates that incentive schemes can change the habitual health-related behaviors that contribute to non-communicable diseases (Jochelson, 2007, Sutherland et al., 2008), uncertainties remain about the conditions under which change is achieved and sustained after incentive removal (Jochelson, 2007, Marteau et al., 2009).

Authors of relevant existing systematic reviews (Jochelson, 2007, Kane et al., 2004, Sutherland et al., 2008, Cahill and Perera, 2011, Paul‐Ebhohimhen and Avenell, 2008) have concluded that achieved changes to habitual health-related behaviors are not sustained after removal of financial incentives. However, these reviews have assessed effects over time, without explicitly focusing on or systematically analysing impacts after incentive removal. This distinction is important since in some studies payment of the final incentive has coincided with the final follow-up assessment (Donatelle et al., 2000a, Donatelle et al., 2000b, Gallagher et al., 2007, Jeffery et al., 1990, Klesges et al., 1987, Rand et al., 1989). Furthermore, most existing systematic reviews have not investigated factors that may modify behavioral responses to incentives, such as the target behavior (Jochelson, 2007, Sutherland et al., 2008), incentive value (Sutherland et al., 2008, Paul‐Ebhohimhen and Avenell, 2008, Lussier et al., 2006), certainty of incentive attainment (certain — e.g. vouchers — vs. uncertain — e.g. lottery) (Leung et al., 2002) and recipients' deprivation level (Sutherland et al., 2008). Some evidence suggests that under the right conditions financial incentives could lead to sustained changes (Cahill and Perera, 2011, Troxel and Volpp, 2012, Volpp et al., 2009), highlighting the need for research to move beyond the question of whether incentives work, to elucidate the circumstances under which they are most effective in achieving and sustaining changes (Marteau et al., 2009).

The present systematic review aims to provide a more complete assessment of the effects of personal financial incentives on habitual health-related behaviors in adults by investigating:

i. the effectiveness of incentives for smoking cessation, healthier eating, reduced alcohol consumption, and increased physical activity,
a. regardless of whether incentives are still offered, and
b. when incentives have been removed;
ii. whether the effectiveness of financial incentives is modified by
a. the target behavior,
b. incentive value and attainment certainty, and
c. recipients' deprivation level.

## Methods

Further information on the review methods are presented in the protocol registered on PROSPERO, record ID CRD42012002675 (available here: http://www.crd.york.ac.uk/PROSPERO/display_record.asp?ID=CRD42012002675).

### Study eligibility criteria

Studies eligible for consideration in this review were randomized controlled trials assessing outcomes relating to target behaviors at a minimum of six months from baseline and allocating adults to the offer of financial incentives or i) no treatment; ii) the same treatment as those incentivized, without the offer of incentives; or iii) incentives differing in attainment certainty or amount. Studies assessing multi-component interventions precluding assessment of the independent effects of incentives, and studies offering incentives of symbolic or no monetary value or not contingent on achievement of target outcomes, were not eligible.

### Literature searches

A detailed search strategy (Appendix A, Text S1) was used to search the following electronic databases for records of eligible studies from inception to July 2012: MEDLINE, EMBASE, PsycINFO, CINAHL, SCOPUS, EconLit, the Cochrane Central Register of Controlled Trials and the Cochrane Database of Systematic Reviews. Searches were limited to studies of adults. No language restrictions were applied. Reference lists of relevant papers and grey literature resources (HMIC, online clinical trials registers, Google Scholar and websites of key organizations) were also searched.

### Study selection and data extraction

The titles and abstracts of identified records were screened by one author (EM). The full-text reports of potentially eligible studies were independently assessed by one author (EM) and one trained research assistant (JT). Disagreements were resolved by consensus.

One author (EM) and one trained research assistant (LSR) independently extracted all data.

Dichotomous outcome data were extracted as measures of effectiveness in terms of the attainment or non-attainment of pre-specified target levels of behavior-change, to allow for overall estimates of behavior-change across target behaviors. If outcome data were unavailable or not presented in dichotomous form, study authors were contacted and requested to provide these. Where these data were unavailable, continuous data were extracted and later re-expressed as odds ratios (see Data analysis). Relevant existing systematic reviews were also checked for missing data.

During the data extraction process, incentives were classified according to their overall value as either ‘low’ (<$400) or ‘high’ (≥$400). Judgments regarding the classification of value for the only study included in the review which was conducted in a low income country (Giné et al., 2010) were made based on information reported by the study authors that incentives constituted approximately 20% of participants' monthly income. Checks conducted using the http://www.usinflationcalculator.com/ website confirmed that the classification of value for all studies included in the analyses remained the same when taking inflation into account. Incentives were also classified according to their type as ‘certain’ (all incentives excluding lotteries) or ‘uncertain’ (lotteries). Participants' deprivation level was classified at the study level as either ‘high’ or ‘other’ based on any relevant information available in the included reports (e.g. income, employment, education, ethnicity, SES scores). If no information was reported to allow classifications to be made, study authors were contacted and requested to provide relevant data (See registered protocol on PROSPERO, available here: http://www.crd.york.ac.uk/PROSPERO/display_record.asp?ID=CRD42012002675 for a justification of the cut-off level regarding the classification of incentive value, as well as the pre-specified conditions relating to the classification of deprivation level).

### Assessment of methodological quality of included studies

One author (EM) and one trained research assistant (LSR) independently assessed the risk of bias of included studies, by applying the Cochrane Collaboration risk of bias tool (Higgins et al., 2011), following definitions and criteria provided in the *Cochrane Handbook for Systematic Reviews of Interventions* (Higgins et al., 2011). When judging the risk of performance bias, the level of standardization of study procedures between groups (i.e. whether studies had controlled for the additional processes inherit in the delivery of incentives, compared to regular treatment) was also assessed. When judging the risk of detection bias, the reliability of outcome measures (i.e. whether outcome assessors could have been deceived by participants) was also assessed. For cluster-randomized controlled trials, the potential risk of recruitment bias was also considered. Inconsistencies in judgements were resolved by consensus.

### Data analysis

Dichotomous outcome data were analysed by calculating an odds ratio (OR) for each study as effect size, along with a 95% confidence interval. Outcomes assessed at various time-points were analyzed separately based on pre-specified intervals and the availability of data corresponding to each of these (months from intervention start: 6, > 6–12, > 12–18, > 18; months from incentive removal: > 2–3, > 3–6, > 6). When dichotomous data were not available, but continuous outcome data were, a standardized mean difference (SMD) was calculated and converted to an odds ratio on the basis of a logistic distributional assumption for the continuous outcome (Anzures‐Cabrera et al., 2011). Specifically, the approximate log(OR) was obtained as $SMD\times \pi /\sqrt{3}$. Missing standard deviations for change in body weight were calculated using the formula proposed by Avenell et al. (2004) (SD of weight change = 5.915 + (0.283 × absolute value of mean change in weight)).

Heterogeneity was assessed via examination of forest plots and calculation of the I-squared statistic. Data were synthesized via meta-analyses grouped by timed endpoints. Univariable and multivariable meta-regressions were conducted to assess the effect of moderating variables on log(OR). These were conducted for outcomes relating to 6 and > 6–12 months from baseline, and > 2–3 and > 6 months from incentive removal, but not for other time-points due to the small number of corresponding between-study comparisons. Moderating variables investigated were target behavior, incentive attainment certainty, incentive value, participant deprivation level, and judgements of potential bias related to standardization of study procedures and reliability of outcome measurements. Two-way interactions were examined between pairs of effect modifiers. All meta-regression analyses were conducted using metareg in Stata (Harbord and Higgins, 2008). Summary effect sizes and their 95% confidence intervals were computed using random-effects meta-analysis models.

## Results

**Fig. 1:**
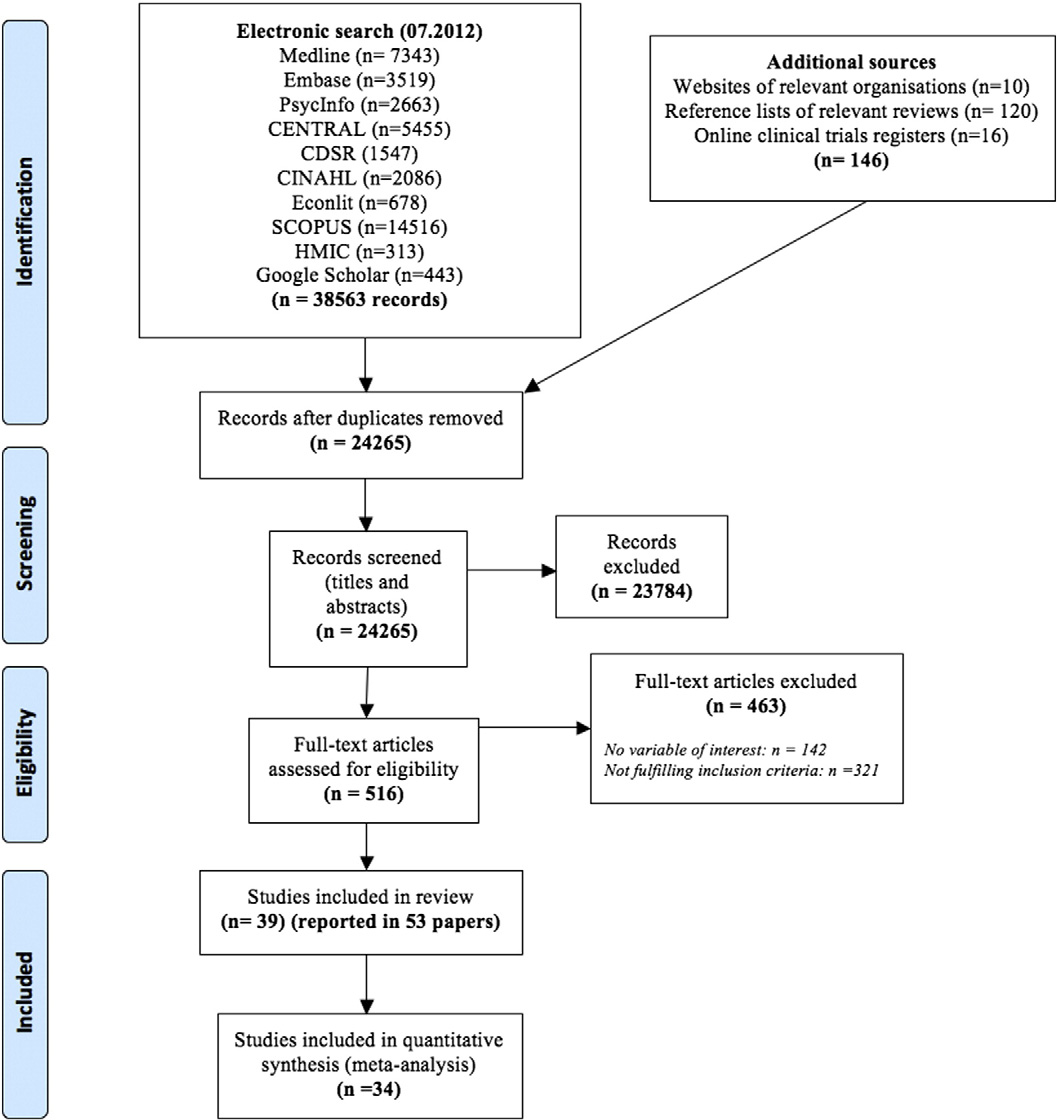
PRISMA flow diagram.

The flow of studies through the systematic review process is presented in Fig. 1 (Moher et al., 2009). From 24,265 unique study records identified by searches, 39 studies (reported in 53 articles) were accepted into the review (Appendix A, Text S2). Thirty-four of these, comprising 10,585 adult participants, were included in the meta-analysis. Five studies were excluded from the analysis for four reasons: report of unsuitable measures of outcome dispersion (Francisco et al., 1994); inclusion of incentivized groups not differing in value and/or attainment certainty without a control group to which a combination of these could be compared (Jeffrey, 1983); lack of data relating to follow-ups at or beyond 6 months from baseline (Mahoney, 1974, Norton and Powers, 1980); use of crossover method in the delivery of incentives and a lack of control group to which a combination of the treatment groups could be compared (Wing et al., 1981). Characteristics and results of included studies are presented in Tables S1 & S2 (Appendix A).

The majority of included studies (n = 36) were conducted in the USA (Donatelle et al., 2000a, Donatelle et al., 2000b, Gallagher et al., 2007, Jeffery et al., 1990, Klesges et al., 1987, Rand et al., 1989, Volpp et al., 2009, Francisco et al., 1994, Jeffrey, 1983, Mahoney, 1974, Norton and Powers, 1980, Wing et al., 1981, Donatelle and Hudson, 2002, Galbo, 2011, Glasgow et al., 1993, Heil et al., 2008, Hennrikus et al., 2002, Higgins et al., 2004, Higgins et al., 2012, Jason et al., 1995, Jeffery et al., 1984, Jeffery et al., 1998, Jeffery et al., 1993, John et al., 2011, Klem and Klesges, 1988, Kramer et al., 1986, Long et al., 2012, Saccone and Israel, 1978, Shoptaw et al., 2002, Volpp et al., 2008, Volpp et al., 2006, Windsor et al., 1988, Wing et al., 1996, Bloch et al., 2006, Crowley et al., 1995, Tevyaw et al., 2009). Twelve were conducted within workplaces (Klesges et al., 1987, Rand et al., 1989, Volpp et al., 2009, Francisco et al., 1994, Galbo, 2011, Glasgow et al., 1993, Hennrikus et al., 2002, Jason et al., 1995, Windsor et al., 1988, Bloch et al., 2006, Gomel et al., 1993, Hunter, 2011), 15 within the community (Jeffery et al., 1990, Giné et al., 2010, Jeffrey, 1983, Mahoney, 1974, Norton and Powers, 1980, Wing et al., 1981, Jeffery et al., 1984, Jeffery et al., 1998, Jeffery et al., 1993, John et al., 2011, Klem and Klesges, 1988, Kramer et al., 1986, Saccone and Israel, 1978, Volpp et al., 2008, Wing et al., 1996), 11 in medical/health settings (Donatelle et al., 2000a, Donatelle et al., 2000b, Gallagher et al., 2007, Donatelle and Hudson, 2002, Heil et al., 2008, Higgins et al., 2004, Higgins et al., 2012, Long et al., 2012, Shoptaw et al., 2002, Volpp et al., 2006, Crowley et al., 1995) and one in an academic setting (Tevyaw et al., 2009). Nineteen focused on smoking cessation (Donatelle et al., 2000a, Donatelle et al., 2000b, Gallagher et al., 2007, Klesges et al., 1987, Rand et al., 1989, Volpp et al., 2009, Giné et al., 2010, Donatelle and Hudson, 2002, Glasgow et al., 1993, Heil et al., 2008, Hennrikus et al., 2002, Higgins et al., 2004, Higgins et al., 2012, Jason et al., 1995, Shoptaw et al., 2002, Volpp et al., 2006, Windsor et al., 1988, Crowley et al., 1995, Tevyaw et al., 2009), 15 on indicators of healthier eating and/or physical activity (i.e. body weight, blood cholesterol, or haemoglobin levels) (Francisco et al., 1994, Jeffrey, 1983, Mahoney, 1974, Norton and Powers, 1980, Wing et al., 1981, Galbo, 2011, Jeffery et al., 1984, Jeffery et al., 1993, John et al., 2011, Klem and Klesges, 1988, Kramer et al., 1986, Long et al., 2012, Saccone and Israel, 1978, Volpp et al., 2008, Bloch et al., 2006) and two on physical activity (Wing et al., 1996, Hunter, 2011). Three studies targeted more than one behaviour (Jeffery et al., 1990, Jeffery et al., 1998, Gomel et al., 1993). No eligible studies were identified in which healthier eating (rather than changes to indicators of this behaviour) was explicitly incentivized. Furthermore, no eligible studies measuring outcomes relating to alcohol consumption were found. Twenty-six studies included assessment of outcomes after incentive removal (Volpp et al., 2009, Giné et al., 2010, Jeffrey, 1983, Mahoney, 1974, Norton and Powers, 1980, Donatelle and Hudson, 2002, Glasgow et al., 1993, Heil et al., 2008, Hennrikus et al., 2002, Higgins et al., 2004, Higgins et al., 2012, Jason et al., 1995, Jeffery et al., 1984, Jeffery et al., 1993, John et al., 2011, Klem and Klesges, 1988, Saccone and Israel, 1978, Shoptaw et al., 2002, Volpp et al., 2008, Volpp et al., 2006, Windsor et al., 1988, Wing et al., 1996, Crowley et al., 1995, Tevyaw et al., 2009, Gomel et al., 1993, Hunter, 2011). The duration of financial incentive schemes ranged from three weeks (Tevyaw et al., 2009) to 18 months (Hennrikus et al., 2002, Jeffery et al., 1998, Jeffery et al., 1993). Most studies (n = 30) offered incentives alongside concurrent intervention components to change target behaviors, (e.g. counselling, self-help manuals, brochures, professional advice, nicotine replacement therapy) (Donatelle et al., 2000a, Donatelle et al., 2000b, Jeffery et al., 1990, Klesges et al., 1987, Rand et al., 1989, Volpp et al., 2009, Giné et al., 2010, Jeffrey, 1983, Mahoney, 1974, Norton and Powers, 1980, Wing et al., 1981, Donatelle and Hudson, 2002, Galbo, 2011, Hennrikus et al., 2002, Jason et al., 1995, Jeffery et al., 1984, Jeffery et al., 1998, Jeffery et al., 1993, John et al., 2011, Klem and Klesges, 1988, Kramer et al., 1986, Saccone and Israel, 1978, Shoptaw et al., 2002, Volpp et al., 2008, Volpp et al., 2006, Windsor et al., 1988, Wing et al., 1996, Crowley et al., 1995, Tevyaw et al., 2009, Gomel et al., 1993). All studies included in the meta-analysis compared incentives with groups receiving the same treatment as incentivized groups without the offer of incentives. Two studies also included “no treatment” control groups, which were excluded from the analysis (Jeffery et al., 1993, Saccone and Israel, 1978). The incentives used in 32 studies were classified as ‘certain’ (Donatelle et al., 2000a, Donatelle et al., 2000b, Gallagher et al., 2007, Jeffery et al., 1990, Klesges et al., 1987, Rand et al., 1989, Volpp et al., 2009, Giné et al., 2010, Jeffrey, 1983, Mahoney, 1974, Norton and Powers, 1980, Wing et al., 1981, Donatelle and Hudson, 2002, Galbo, 2011, Heil et al., 2008, Higgins et al., 2004, Higgins et al., 2012, Jason et al., 1995, Jeffery et al., 1984, Jeffery et al., 1998, Jeffery et al., 1993, John et al., 2011, Klem and Klesges, 1988, Kramer et al., 1986, Long et al., 2012, Saccone and Israel, 1978, Shoptaw et al., 2002, Volpp et al., 2006, Windsor et al., 1988, Bloch et al., 2006, Tevyaw et al., 2009, Hunter, 2011). Those used in four studies were classified as ‘uncertain’ (Francisco et al., 1994, Hennrikus et al., 2002, Wing et al., 1996, Crowley et al., 1995) and in two as ‘certain and uncertain’ (i.e. participants were offered vouchers/cash and chances to win lotteries) (Hennrikus et al., 2002, Gomel et al., 1993). One study (Volpp et al., 2008) included two groups differing in incentive attainment certainty. The value of incentives used in 20 studies was classified as ‘low’ (Donatelle et al., 2000a, Jeffery et al., 1990, Klesges et al., 1987, Rand et al., 1989, Francisco et al., 1994, Jeffrey, 1983, Mahoney, 1974, Norton and Powers, 1980, Wing et al., 1981, Jason et al., 1995, Jeffery et al., 1984, Klem and Klesges, 1988, Kramer et al., 1986, Long et al., 2012, Saccone and Israel, 1978, Volpp et al., 2006, Windsor et al., 1988, Bloch et al., 2006, Tevyaw et al., 2009, Hunter, 2011) and as ‘high’ in 18 studies (Donatelle et al., 2000b, Gallagher et al., 2007, Volpp et al., 2009, Giné et al., 2010, Higgins et al., 2011, Galbo, 2011, Glasgow et al., 1993, Heil et al., 2008, Hennrikus et al., 2002, Higgins et al., 2004, Jeffery et al., 1998, Jeffery et al., 1993, John et al., 2011, Shoptaw et al., 2002, Volpp et al., 2008, Wing et al., 1996, Crowley et al., 1995, Gomel et al., 1993). One study (Donatelle and Hudson, 2002) included two incentivized groups differing in their classification of value. Participants' deprivation level was classified as ‘high’ in 12 studies (Donatelle et al., 2000a, Donatelle et al., 2000b, Gallagher et al., 2007, Rand et al., 1989, Giné et al., 2010, Donatelle and Hudson, 2002, Heil et al., 2008, Higgins et al., 2004, Higgins et al., 2012, Shoptaw et al., 2002, Volpp et al., 2006, Crowley et al., 1995) and as ‘other’ in 22 (Jeffery et al., 1990, Klesges et al., 1987, Volpp et al., 2009, Francisco et al., 1994, Jeffrey, 1983, Galbo, 2011, Glasgow et al., 1993, Hennrikus et al., 2002, Jason et al., 1995, Jeffery et al., 1984, Jeffery et al., 1998, Jeffery et al., 1993, John et al., 2011, Kramer et al., 1986, Long et al., 2012, Saccone and Israel, 1978, Volpp et al., 2008, Windsor et al., 1988, Bloch et al., 2006, Tevyaw et al., 2009, Gomel et al., 1993, Hunter, 2011). Five studies did not include any information to allow for the latter classification to be made (Mahoney, 1974, Norton and Powers, 1980, Wing et al., 1981, Klem and Klesges, 1988, Wing et al., 1996)

### Quality of included studies

Reviewers' risk of bias judgements are presented in Fig. S1 (Appendix A).

Most studies provided insufficient detail to enable assessment of the integrity of randomization (n = 26) (Donatelle et al., 2000a, Donatelle et al., 2000b, Klesges et al., 1987, Rand et al., 1989, Francisco et al., 1994, Jeffrey, 1983, Mahoney, 1974, Norton and Powers, 1980, Wing et al., 1981, Donatelle and Hudson, 2002, Galbo, 2011, Glasgow et al., 1993, Heil et al., 2008, Hennrikus et al., 2002, Higgins et al., 2012, Jason et al., 1995, Jeffery et al., 1984, Jeffery et al., 1998, Jeffery et al., 1993, Klem and Klesges, 1988, Kramer et al., 1986, Saccone and Israel, 1978, Wing et al., 1996, Bloch et al., 2006, Tevyaw et al., 2009, Gomel et al., 1993) and allocation concealment (n = 30) (Donatelle et al., 2000a, Donatelle et al., 2000b, Jeffery et al., 1990, Klesges et al., 1987, Rand et al., 1989, Giné et al., 2010, Francisco et al., 1994, Jeffrey, 1983, Mahoney, 1974, Norton and Powers, 1980, Wing et al., 1981, Donatelle and Hudson, 2002, Galbo, 2011, Glasgow et al., 1993, Heil et al., 2008, Hennrikus et al., 2002, Higgins et al., 2012, Jason et al., 1995, Jeffery et al., 1984, Jeffery et al., 1998, Jeffery et al., 1993, John et al., 2011, Klem and Klesges, 1988, Kramer et al., 1986, Saccone and Israel, 1978, Shoptaw et al., 2002, Wing et al., 1996, Bloch et al., 2006, Tevyaw et al., 2009, Gomel et al., 1993). Because of the nature of financial incentive schemes, participants were not blinded in any of the studies. Most did not blind personnel and provided insufficient detail to judge whether this resulted in increased risk of bias (n = 32) (Donatelle et al., 2000a, Donatelle et al., 2000b, Gallagher et al., 2007, Klesges et al., 1987, Rand et al., 1989, Volpp et al., 2009, Giné et al., 2010, Francisco et al., 1994, Jeffrey, 1983, Mahoney, 1974, Norton and Powers, 1980, Wing et al., 1981, Galbo, 2011, Glasgow et al., 1993, Heil et al., 2008, Higgins et al., 2004, Higgins et al., 2012, Jason et al., 1995, Jeffery et al., 1984, Jeffery et al., 1998, Jeffery et al., 1993, John et al., 2011, Klem and Klesges, 1988, Kramer et al., 1986, Saccone and Israel, 1978, Shoptaw et al., 2002, Volpp et al., 2008, Windsor et al., 1988, Wing et al., 1996, Crowley et al., 1995, Tevyaw et al., 2009, Gomel et al., 1993). The majority of studies had sufficiently standardized study procedures between incentivized and control groups, therefore diminishing the possibility that obtained outcomes were the result of the additional processes inherit in incentive delivery (n = 29) (Jeffery et al., 1990, Klesges et al., 1987, Rand et al., 1989, Francisco et al., 1994, Jeffrey, 1983, Norton and Powers, 1980, Wing et al., 1981, Donatelle and Hudson, 2002, Heil et al., 2008, Hennrikus et al., 2002, Higgins et al., 2004, Higgins et al., 2012, Jason et al., 1995, Jeffery et al., 1984, Jeffery et al., 1998, Jeffery et al., 1993, Klem and Klesges, 1988, Long et al., 2012, Saccone and Israel, 1978, Shoptaw et al., 2002, Volpp et al., 2006, Windsor et al., 1988, Wing et al., 1996, Bloch et al., 2006, Crowley et al., 1995, Tevyaw et al., 2009, Gomel et al., 1993, Hunter, 2011). In all but two studies (Volpp et al., 2009, Jeffery et al., 1998), outcome assessors were considered to have been adequately blinded or the risk of bias resulting from a lack of blinding was judged to be minimal. Most studies used reliable outcome measures (n = 27) (Donatelle et al., 2000a, Donatelle et al., 2000b, Gallagher et al., 2007, Volpp et al., 2009, Giné et al., 2010, Francisco et al., 1994, Mahoney, 1974, Norton and Powers, 1980, Galbo, 2011, Glasgow et al., 1993, Heil et al., 2008, Higgins et al., 2004, Higgins et al., 2012, Jeffery et al., 1984, Jeffery et al., 1993, John et al., 2011, Klem and Klesges, 1988, Long et al., 2012, Saccone and Israel, 1978, Shoptaw et al., 2002, Volpp et al., 2008, Volpp et al., 2006, Windsor et al., 1988, Bloch et al., 2006, Crowley et al., 1995, Tevyaw et al., 2009, Gomel et al., 1993). One cluster-randomized controlled trial was considered at high risk of recruitment bias (Giné et al., 2010).

### Impact of financial incentives on habitual health-related behaviours

**Fig. 2:**
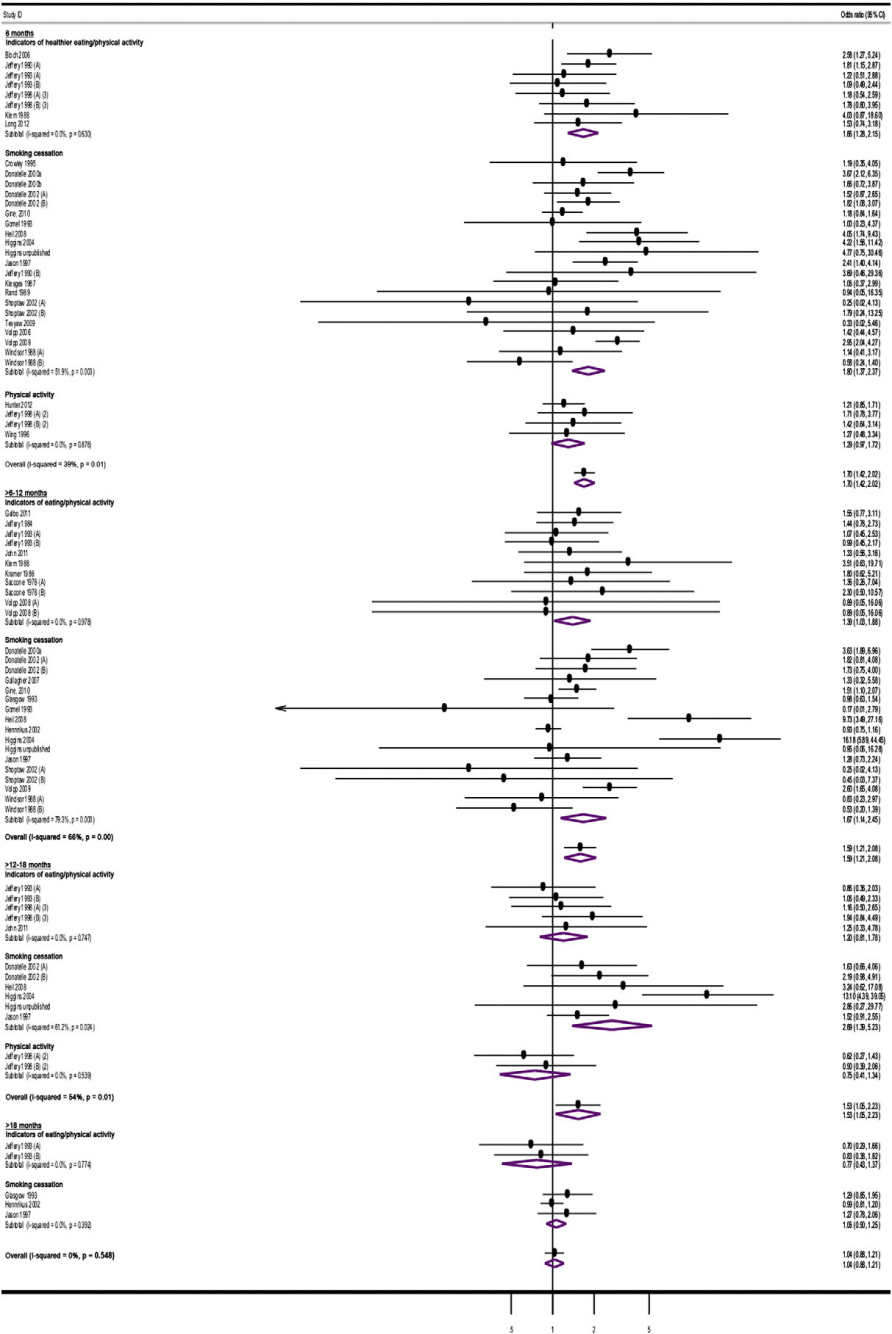
Study estimates of financial incentives effects on health behaviors at time-points from intervention start.

**Fig. 3:**
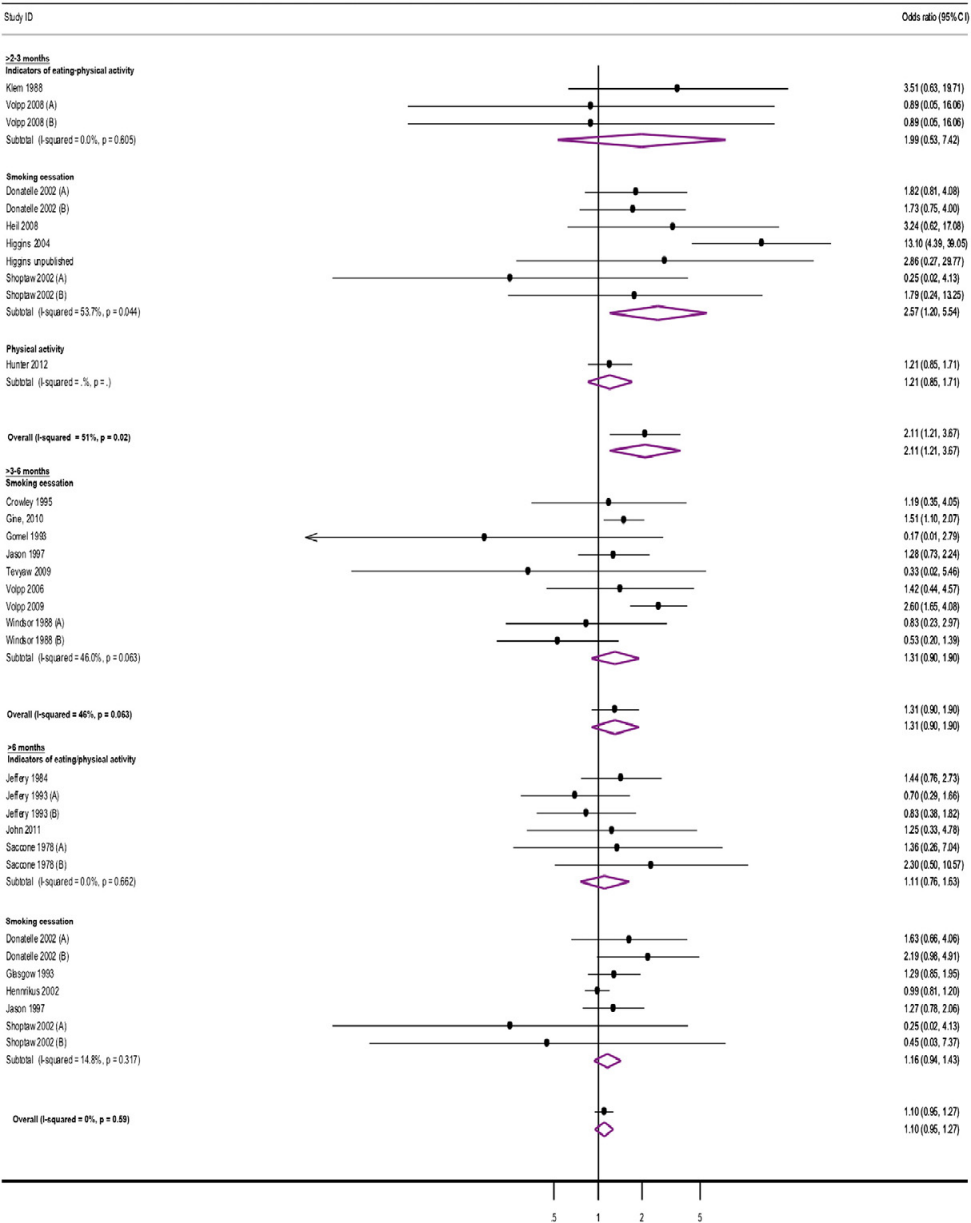
Study estimates of financial incentives effects on health behaviors at time-points after incentive removal.

Moderate levels of heterogeneity were identified in the results of meta-analyses between studies at most time-points, apart from > 18 months from intervention start and > 3–6 and > 6 months from incentive removal. These derived from moderate to substantial inconsistencies observed at these time-points in relation to the results of studies assessing smoking cessation (Fig. 2, Fig. 3).

Personal financial incentives increased attainment of target levels of behavior-change at all time-points from interventions start, apart from > 18 months (Table 1; Fig. 2). Financial incentives were effective in sustaining changes to overall behavior for up to > 2–3 months after incentive removal, but not thereafter (Table 1; Fig. 3). Overall effects across behaviors followed a monotonic trend, weakening over time, when assessed both from intervention start and after incentive removal.

Personal financial incentives increased smoking cessation up to > 12–18 months from intervention start. Improved cessation rates were sustained for up to > 2–3 months after incentive removal. Incentives also increased the attainment of target indicators of healthier eating and/or physical at 6 and > 6–12 months from intervention start. Changes were not sustained after incentive removal. Physical activity was measured only at 6 and > 12–18 months from intervention start and > 2–3 months after incentive removal: financial incentives did not to lead to increased target levels of physical activity at any of these time-points (Table 1, Fig. 2, Fig. 3).

### Effect modifiers

**Fig. 4:**
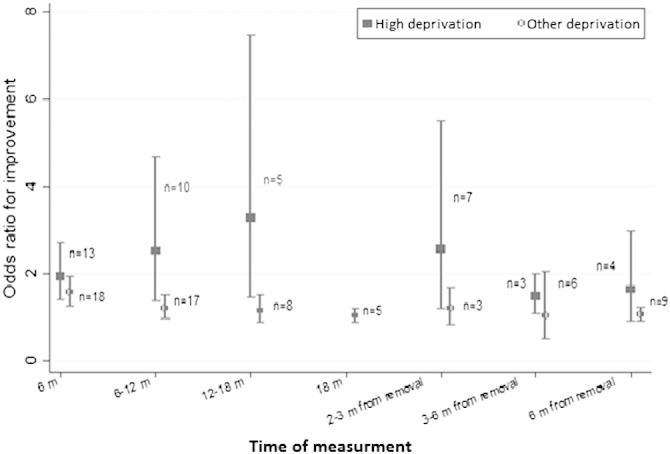
The effect of financial incentives on health-behavior according to recipients' deprivation level at multiple measurement times.

The effect of financial incentives was not independently modified by the target behavior, incentive value or attainment certainty at any of the assessed time-points. Both univariable (Table 2) and multivariable meta-regressions (Appendix A, Table S3) produced similar results. Univariable but not multivariable analysis showed participants' deprivation level modified the effect of incentives at > 6–12 months from intervention start, but not at other time-points: studies including highly deprived participants (n = 10) generated an average effect approximately twice the size of studies including non-deprived participants (n = 10) (OR = 2.17; 95% CI 1.22 to 3.85) (Table 2; Fig. 4).

One interaction was found to be statistically significant at the 5% level at 6 months from intervention start: use of high value incentives was associated with a higher increase in smoking cessation than lower value incentives. The summary odds ratio for smoking cessation from studies using low value incentives (n = 10) was 1.49, CI 95% 1.12 to 1.98. We did not identify any statistically significant two-way interactions at any other time-points.

## Discussion

Personal financial incentives were effective in increasing attainment of target levels of health-related behavior-change, with beneficial effects lasting up to 18 months from intervention start, but weakening over time. Changes were sustained up to three months after incentive removal. The target behavior, incentive value and attainment certainty did not independently modify effects at any time-point. An interaction between target behavior and incentive value modified effects at six months from intervention start, with high value incentives increasing smoking cessation. Recipients' deprivation level modified effects between six and 12 months from intervention start, with higher deprivation increasing attainment of target levels of behavior-change.

To our knowledge, this is the first systematic review to provide an overall estimate of the impact of financial incentives across habitual health-related behaviors. Interpretation of the findings, however, requires some caution. Not all behaviors classified as habitual and health-related were represented in this review. Searches did not yield any eligible studies assessing outcomes related to alcohol consumption. Furthermore, although the overall effectiveness of incentives weakened over time, this coincided with a decrease in the number of comparisons at each time-point. As such, findings might represent a true negative effect or limited statistical power to detect effects at later time-points. Moreover, although incentive effectiveness was not modified by the target behavior at any time-point, inspection of impacts on individual behaviors suggests that summary effect sizes were driven by studies assessing smoking cessation: This was the only behavior for which changes were maintained up to 18 months from intervention start and sustained after incentive removal. Finally, although attainment of target levels of physical activity was unaffected by the offer of financial incentives, firm conclusions would be premature given that only three included studies assessed physical activity.

Most eligible studies included in this systematic review evaluated interventions targeting smoking cessation, which could partially explain why incentives appear more promising for changing this behavior compared with others. A novel finding of this review, permitted by an explicit focus on post-incentive effects, is that smoking cessation was sustained after incentive removal. This may be in part related to inclusion in this review of studies assessing the impact of financial incentives on smoking cessation during pregnancy (Donatelle et al., 2000a, Donatelle et al., 2000b, Donatelle and Hudson, 2002, Heil et al., 2008, Higgins et al., 2004, Higgins et al., 2012). Indeed, five out of seven comparisons assessing smoking cessation > 2–3 months after incentive removal targeted pregnant smokers. Personal financial incentives are the most effective intervention for smoking cessation in pregnancy (Bauld and Coleman, 2009, Lumley et al., 2009), although the sustainability of their effects remains unexplored. One of the characteristics of studies demonstrating this effectiveness is the use of large rewards. Large rewards have been predicted to motivate greater behavior-change (Jochelson, 2007, Sutherland et al., 2008, Lussier et al., 2006, Sigmon and Patrick, 2012). Incentive value in the present review modified the impact of incentives on smoking cessation at six months from intervention start, but not after incentive removal or at other time-points. Perhaps the classification of incentive value in this review was too crude to allow for effects to be detected at other time-periods, or there was insufficient statistical power to do so. Alternatively, if the sustained effects of incentives on smoking cessation reported herein are related to the inclusion of studies incentivizing pregnant smokers, then perhaps the key variable is not only incentive value, but also some of the other specific incentive scheme characteristics of these studies (such as the use of high frequency incremental reinforcement schedules that become gradually less frequent overt time (Marteau et al., 2013)), the role of which was not assessed in this review.

Although findings show that smoking cessation can be sustained, the evidence suggests that this effect does not persist beyond three months after incentive removal. Between three and six months from incentive removal only two studies out of nine significantly favored the use of incentives (Volpp et al., 2009, Giné et al., 2010). The ability of one of these to detect effects (Volpp et al., 2009) has been attributed to the use of a large sample size and large rewards (Cahill and Perera, 2011, Troxel and Volpp, 2012), characteristics shared by both these studies. There was insufficient power to conduct meta-regression analyses at this time-point to determine whether under some conditions improvements could be sustained beyond three months. The lack of significant effects and effect modifiers beyond six months from removal however, suggests that ultimately changes disappear, regardless of the circumstances surrounding incentive delivery.

In interpreting the effects of personal financial incentives on indicators of healthier eating and/or physical activity, it should be noted that outcomes assessed beyond six months from intervention start relate to weight-loss. Consistent with the findings from a previous meta-analysis (Paul‐Ebhohimhen and Avenell, 2008), incentives did not improve weight-loss beyond 12 months from intervention start and changes were not sustained after incentive removal. The reduced duration of incentive effects on weight-loss compared to smoking cessation might have several explanations. First, change in body weight reflects the cumulative effects of many behaviors over time rather than the effect of a single behavior (Jeffery, 2012). Second, many of the studies on weight-loss included in the current review had small sample sizes. This, in combination with the reported weakening of incentive effects over time, might have resulted in a lack of power to detect effects at later time-points. Finally, whereas the majority of studies on smoking cessation used rewards, most studies on weight-loss used deposit contracts. Requiring individuals to pledge their own funds rather than directly reinforcing them might differentially affect outcomes. The moderating effect of this incentive characteristic was not assessed in the present review.

Financial incentives have been predicted to be more effective in motivating behavior-change in the most deprived (Sutherland et al., 2008). Consistent with this, between six and 12 months from intervention start, the effect of incentives across habitual health-related behaviors was found to be greater for those classified as highly deprived. This is the first empirical evidence that we are aware of demonstrating the role of recipients' deprivation level in the moderation of the impact of financial incentives on health-related behavior. It is an important finding as it suggests that incentive schemes contribute to reducing health inequalities. Although impacts appeared greater for highly deprived individuals at all time-points, differences were significant only at one of these.

The effectiveness of financial incentives has also been suggested to vary according to whether incentive attainment is certain (e.g. voucher or cash payment) or uncertain (e.g. a lottery ticket), with some studies suggesting the former to be more effective (Leung et al., 2002, Niza et al., 2014). Findings from this review, however, suggest that changes to habitual health-related behaviors are unaffected by the certainty of incentive attainment. Given that the incentives of very few of the included studies were classified as ‘uncertain’ (Hennrikus et al., 2002, Volpp et al., 2008, Wing et al., 1996, Crowley et al., 1995), it would be premature to draw conclusions from this review regarding the moderating role of this incentive characteristic.

### Implications

Behavior-change maintenance is critical for reducing the burden of non-communicable diseases and should be the aim of interventions targeting health-related behavior-change. Although the use of personal financial incentives appears useful in initiating healthier behaviors, with changes sustained for some months after incentive removal, results from this review suggest that effects may ultimately dissipate, i.e. new habits do not appear to be formed. This is a problem shared by most interventions targeting habitual health-behaviors at an individual level (Ogden, 2012). This review did not compare the use of incentives with other behavior-change strategies. It is therefore unknown whether incentives are better than other strategies at producing short-term changes. If so, it might be worth complementing their use with behavior-maintenance and relapse-prevention strategies, which could be delivered after incentive removal. Consideration of the use of financial incentive schemes, however, should be informed by formal cost-effectiveness analyses, which so far are rare. Even if cost-effective, their application will depend on their acceptability to policy makers, health professionals and the public. Their use for health promotion attracts negative views (Promberger et al., 2011), although these can be attenuated by evidence of their effectiveness (Promberger et al., 2012). Consequently, what is found effective in studies will not necessarily be considered acceptable in practice (Volpp et al., 2011).

Given the lack of sustained effects, in addition to the costs and compromised acceptability of using financial incentives for changing health-related behaviors, future research and policies should consider the application of financial mechanisms in the context of policies that alter environments at a population level, making health behavior more likely to be sustained (Marteau et al., 2012).

### Strengths

This is the first systematic review to provide an overall estimate of the impact of personal financial incentives across a range of habitual health-related behaviors. It is also the first to focus explicitly on assessment of the sustained effects of incentives on habitual health-related behaviors after their removal. Furthermore, it is one of the few reviews to systematically assess the role of potential effect modifiers, thus attempting to elucidate the circumstances under which incentives are most effective. It is also the first research that we are aware of to demonstrate the role of recipient deprivation level in moderating the effects of financial incentives on health-related behavior, thus highlighting the potential of incentive schemes to reduce health inequalities.

### Limitations

The main limitation of this review is the small number of studies and associated lack of statistical power for certain comparisons, which restricts the conclusions that can be drawn with regard to: i. the sustained impact of financial incentives on overall behavior beyond 18 months from intervention start; ii. the impact of financial incentives on physical activity; and iii. the role of certain of the targeted effect modifiers. Furthermore, given the lack of eligible studies on alcohol consumption, findings cannot be applied to all habitual health-related behaviors. In addition, the roles of many other potential effect modifiers, such as whether the incentive schemes involved the use of deposit contracts, were not examined. Finally, a further minor limitation is that we were unable, within available resources, to allocate more than one person to screen title and abstract records in order to exclude records of clearly ineligible studies prior to full-text screening. However, two reviewers did work independently, blinded to each other's initial decisions, to screen corresponding full-text study reports, which were used as the basis for final inclusion decisions.

## Conclusion

Personal financial incentives change habitual health-behaviors and may help reduce health inequalities. However, their role in reducing non-communicable disease burden is potentially limited, given the current evidence that effects are not sustained beyond three months after incentive removal.

## Funding disclosure

This research was funded by the Wellcome Trust as part of a Strategic Award in Biomedical Ethics; program title: “The Centre for the Study of Incentives in Health”; grant number: 086031/Z/08/Z; PI Prof. TM Marteau. The funder did not contribute to any part of this research.

## Author contributions

*Study concept and design*: EM, FV and TMM conceived and designed the systematic review and meta-analysis in consultation with IS.

*Acquisition of data*: EM

*Statistical analysis*: YW, and JPTH

*Statistical analysis*: YW and JPTH

*Interpretation of data*: EM, FV, TMM, IS, YW and JPTH

*Draft of the manuscript*: EM with input from FV, TMM, IS, YW and JPTH

All authors had full access to all of the data (including statistical reports and tables) in the study and can take responsibility for the integrity of the data and the accuracy of the data analysis

## Ethical approval

Ethical approval not needed.

## Conflict of interest statement

All authors declare no conflicts of interests. Specifically, they declare: no support from any organization for the submitted work; no financial relationships with any organizations that might have an interest in the submitted work in the previous three years; and no other relationships or activities that could appear to have influenced the submitted work.

## Figures and Tables

**Table 1:**
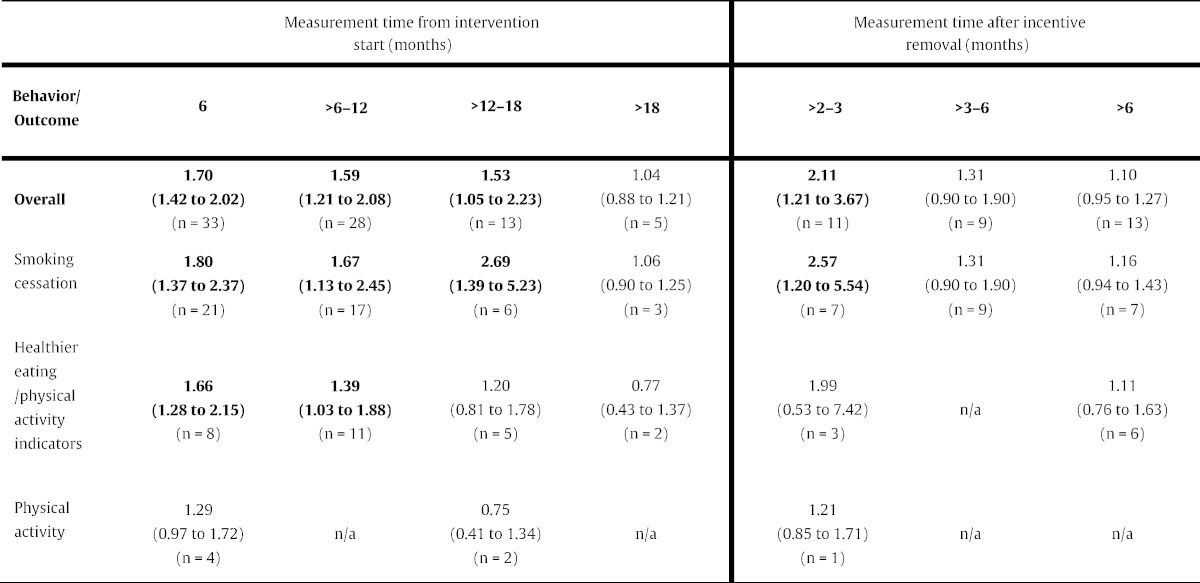
Overall behavior-change (summary odds ratio with 95% CI) and change for targeted behaviors.

Note: n denotes number of comparisons. Eight studies (Jeffery et al., 1990, Donatelle and Hudson, 2002, Jeffery et al., 1998, Jeffery et al., 1993, Saccone and Israel, 1978, Shoptaw et al., 2002, Volpp et al., 2008, Windsor et al., 1988) included more than one incentivized group and appropriate control and thus offered more than one comparison at assessed time-points. These were included in the analysis as separate studies.

**Table 2:** Results from meta-regression analyses according to time-point.

	Univariable meta-regression							
	Measurement time from intervention start (months)				Measurement time after incentive removal (months)			
**Behavior**	Coefficient estimates (95% CI)	P-values	Coefficient estimates (95% CI)	P-values	Coefficient estimates (95% CI)	P-values	Coefficient estimates (95% CI)	P-values
Smoking cessation vs. healthier eating/physical activity indicators	0.73 (0.44 to 1.23) (n = 21 vs. 8)	0.23	0.85 (0.44 to 1.65) (n = 17 vs. 11)	0.63	0.70 (0.09 to 6.18) (n = 7 vs. 3)	0.70	0.95 (0.57 to 1.60) (n = 7 vs. 6)	0.83
Smoking cessation vs. physical activity	0.90 (0.59 to 1.37) (n = 21 vs. 4)	0.60	n/a (n = 17 vs 0)	n/a	0.47 (0.08 to 2.87) (n = 7 vs. 1)	0.36	n/a (n = 7 vs. 0)	-
**Attainment certainty**								
Certain vs. uncertain	0.57 (0.11 to 3.05) (n = 30 vs. 2)	0.46	0.53 (0.16 to 1.69) (n = 24 vs. 2)	0.27	0.41 (0.01 to 16.65) (n = 10 vs. 1)	0.60	0.78 (0.54 to 1.14) (n = 11 vs. 1)	0.18
Certain vs. certain and uncertain	0.71 (0.28 to 1.80) (n = 30 vs. 2)	0.51	0.44 (0.13 to 1.48) (n = 24 vs. 2)	0.18	n/a (n = 10 vs. 0)	n/a	1.02 (0.58 to 1.79) (n = 11 vs. 1)	0.94
**Monetary value**								
High vs. low	0.84 (0.58 to 1.22) (n = 18 vs. 15)	0.35	0.81 (0.41 to 1.58) (n = 19 vs. 9)	0.52	0.66 (0.18 to 2.48) (n = 8 vs. 3)	0.50	1.36 (0.89 to 2.07) (n = 8 vs. 6)	0.14
**Level of deprivation**								
High vs. low	1.25 (0.84 to 1.87) (n = 18 vs. 13)	0.26	**2.17 (1.22 to 3.85) (n = 17 vs. 10)**	**0.01**	2.32 (0.50 to 10.71) (n = 3 vs 7)	0.24	1.55 (0.79 to 3.03) (n = 9 vs. 4)	0.18
**Procedure standardization bias**								
Low vs. high	1.33 (0.85 to 2.08) (n = 29 vs. 4)	0.13	1.09 (0.57 to 2.07) (n = 18 vs. 10)	0.89	0.40 (0.03 to 5.71) (n = 8 vs. 2)	0.45	1.16 (0.68 to 1.98) (n = 9 vs. 4)	0.56
**Outcome measure reliability bias**								
Low vs. high	0.90 (0.58 to 1.39) (n = 24 vs. 7)	0.62	0.65 (0.24 to 1.76) (n = 24 vs. 2)	0.38	0.39 (0.07 to 2.09) (n = 8 vs. 1)	0.23	0.88 (0.60 to 1.29) (n = 9 vs. 2)	0.48
Low vs. unclear	0.95 (0.50 to 1.82) (n = 24 vs 2)	0.87	1.07 (0.35 to 3.31) (n = 24 vs 2)	0.90	0.58 (0.13 to 2.58) (n = 8 vs 2)	0.42	1.66 (0.78 to 3.54) (n = 9 vs 2)	0.17

Note: n denotes number of comparisons.
