# Supplementary material for: Personal financial incentives for changing habitual health-related behaviors: A systematic review and meta-analysis
Source: Prev Med. 2015 Jun;75:75–85. doi: 10.1016/j.ypmed.2015.03.001 (PMC4728181; doi:10.1016/j.ypmed.2015.03.001)
Supplement: Supplementary file 1 — Supplementary material. [file mmc1.docx]

# Personal financial incentives for changing habitual health-related behaviors: a systematic review and meta-analysis.

**Appendix A –Supplementary data**

Table of contents

Supplementary [Text 2](#_Toc368139485)

[Text S1. MEDLINE (Ovip SP) Search strategy 2](#_Toc368139485)

[Text S2. Studies included in review 7](#_Toc368139486)

[Supplementary Figures 12](#_Toc368139487)

[Figure S1. Risk of bias summary 12](#_Toc368139487)

[Supplementary Tables 13](#_Toc368139488)

[[Table S1. Characteristics of included studies 13](#_Toc368139488)](#_Toc368139488)

[Tables S2. Results of included studies 37](#_Toc368139488)

[Tables S3. Multivariate analyses 44](#_Toc368139488)

#

**Supplementary Text**

**Text S1: MEDLINE (Ovid SP) Search Strategy**

**1.** exp Smoking OR Smoking.mp

**2.**  exp Smoking Cessation OR smoking cessation.mp

**3.** exp Tobacco Use Cessation

**4.** (quit* adj3 smok*).mp

**5.** (smok* adj3 abstinen*).mp

**6.** (cut* down adj3 cigarette*).mp

**7.** (smok* adj3 reduc*).mp

**8.** (cigarette* adj3 reduc*)

**9.** (CO adj3 reading*).mp

**10.** (CO adj3 level*).mp

**11.** (carbon monoxide adj3 reading*)

**12.**  (carbon monoxide adj3 level*) OR cotinine adj3 level*.mp OR nicotine adj3 addict*

**13.** tobacco.mp OR exp Tobacco)

**14.** 1 OR 2 OR 3 OR 4 OR 5 OR 6 OR 5 OR 8 OR 9 OR 10 OR 11 OR 12 OR 13

**15.** exp Exercise **OR** exercise.mp

**16.** (physical adj3 exercis*).mp

**17.** (physical adj3 train*).**mp**

**18.** (physical adj3 activ*).mp

**19.** (physical adj3 inactiv*).mp

**20.** (sedentary adj3 behavio?r*).mp **OR** (sedentary adj3 lifestyle*).mp

**21.** (sedentary adj3 life-style*).

**22.** (sedentary behavio?r* adj3 modif*).mp

**23.** (sedentary lifestyle* adj3 modif*).mp

**24.** (sedentary life-style* adj3 modif*).

**25.** exp Physical Education and Training

**26.** gym*.mp

**27.** exp Sports

**28.** (gym* adj3 attend*).mp

**29.** exp Walking

**30.** exp Running

**31.** exp Jogging

**32.** fitness.mp

**33.** exp Physical Fitness

**34.** walk*

**35.** run*

**36.** jog*

**37.** (aerobic* adj3 exercis*)

**38.** (aerobic* adj3 activit*)

**39.** (aerobic* adj3 train*)

**40.** cardiorespiratory adj1 fitness.mp

**41.** exp Swimming

**42.** swim*.mp

**43.** 15 OR 16 OR 17 OR 18 OR 19 OR 20 OR 21 OR 22 OR 23 OR 24 OR 25 OR 26 OR 27 OR 28 OR 29 OR 30 OR 31 OR 32 OR 33 OR 34 OR 35 OR 36 OR 37 OR 38 OR 39 OR 40 OR 41 OR 42

**44.** exp Diet **OR** diet*

**45.** Diet therapy

**46.** exp Food

**47.** exp Beverages

**48.** nutrition.mp

**49.** exp Nutrition assessment

**50.** (nutrition* adj3 choice*).mp

**51.** (nutrition* adj3 preference*).mp

**52.** (healthy adj3 eating).mp

**53.** (healthy adj3 diet).mp

**54.** (diet* adj3 preference*).mp

**55.** (diet* adj3 behavio?r*).mp

**56.** (food* adj3 preference*).mp

**57.** (food* adj3 choice*).mp

**58.** (diet* adj3 choice*).mp

**59.** (nutrition* adj3 habit*).mp

**60.** (diet* adj3 habit*).mp

**61.** (diet* adj3 modif*).mp

**61.** (food* adj3 habit*)

**62.** (eat* adj3 habit*).mp

**63. (**food* adj3 consum*).mp

**64.** (vegetable* adj3 consum*).mp

**65.** (vegetable* adj3 intake*).mp

**66.** (fruit* adj3 consum*).mp

**67.** (fruit* adj3 intake*).mp

**68.** (beverage* adj3 consum*)

**69.** (fat* adj3 consum*).mp

**70.** (fat* adj3 intake*)

**71.** weight-loss.mp

**72.** weight loss mp.

**73.** exp Weight Loss

**74.** (weight adj3 decrease*).mp

**75.** (weight adj3 reduc*).mp

**76.** (calorie* adj3 intake*).mp

**77.** (calorie* adj3 consum*).mp

**78.** (calorie* adj3 decrease).mp

**79.** (calorie* adj3 reduc*).mp

**80.** (calorie* adj3 cut*down).mp

**81.** (calorie* adj3 control*).mp

**82.** (fat* adj3 reduc*).mp

**83.** (fat* adj3 decrease*).mp

**84.** (fat* adj3 loss*).mp

**85.** exp Obesity

**86.** exp Body Mass Index

**87.** exp Body Weight

**88.** body weight.mp

**89.** BMI.mp

**90.** exp Overweight

**91.** exp Obesity, Morbid

**92.** obes*.mp

**93.** exp Feeding Behavior

**94.** overweight.mp

**95.** (weight adj3 control*).mp

**96.** (waist-hip adj1 ratio).mp

**97.** (skinfold adj1 thickness).mp

**98.** (obesity adj3 prevent*).mp

**99.** exp Eating

**100.** exp Hypherphagia OR hyperphagia.mp

**101.**overeat*.mp

**102.** exp Energy Intake

**103.** energy intake.mp

**104.** (over eat*).mp

**105.** overfeed*.mp

**106.** (over feed*).mp

**107.** exp Overnutrition OR overnutruition.mp

**108.** adipose.mp

**109.** exp Adipose Tissue

**110.** (fat * adj3 content).mp

**111.** (fat * adj3 distribut*).mp

**112.** cholesterol adj3 blood

**113.** glucose adj3 blood

**114.** 44 OR 45 OR 46 OR 47 OR 48 OR 49 OR 50 OR 51 OR 52 OR 53 OR 54 OR 55 OR 56 OR 57 OR 58 OR 59 OR 60 OR 61 OR 62 OR 63 OR 64 OR 65 OR 66 OR 67 OR 68 OR 69 OR 70 OR 71 OR 72 OR 73 OR 74 OR 75 OR 76 OR 77 OR 78 OR 79 OR 80 OR 81 OR 82 OR 83 OR 84 OR 85 OR 86 OR 87 OR 88 OR 89 OR 90 OR 91 OR 92 OR 93 OR 94 OR 95 OR 96 OR 97 OR 98 OR 99 OR 100 OR 101 OR 102 OR 103 OR 104 OR 105 OR 106 OR 107 OR 108 OR 109 OR 110 OR 111 OR 112 OR 113

**115.** exp Alcohol Drinking/

**116.** alcohol*.mp.

**117.** alcohol reduction.mp.

**118.** alcohol therapy.mp.

**119.** Alcohol intervention*.mp.

**120.** (alcohol* adj1 use*).mp.

**121.** (alcohol* adj1 abuse*).mp.

**122.** (alcohol* ad1 misuse*).mp.

**123.** (binge* adj1 drink*).mp.

**124.** (alcohol* adj1 problems*).mp.

**125.** binge drink*.mp.

**126.** alcohol use*.mp.

**127.** alcohol abuse*.mp.

**128.** alcohol misuse*.mp.

**129.** 115 OR 116 OR 117 OR 118 OR 119 OR 120 OR 121 OR 122 OR 123 OR 124 OR 125 OR 126 OR 127 OR 128

**130.** 14 OR 43 OR 114 OR 129

**131.**Incentive*.mp

**132.** exp Motivation

**133.** exp Reimbursement, Incentive

**134.** (financial adj3 incentive*).mp

**135.** (pay* adj3 incentive*).mp

**136.** (cash adj3 incentive*).mp

**137.** (money adj3 incentive*).mp

**138.** (monetary adj3 incentive*).mp

**139.** (economic adj3 incentive*).mp

**140.** exp Reward OR reward*

**141.** pay*

**142.** prize*.mp

**143.** award*.mp

**144.** cash.mp

**145.** money.mp

**146.** monetary.mp

**147.** (cash adj3 pay*).mp

**148.** (cash adj3 transfer*).mp

**149.** (contingen* adj3 contract*.mp)

**150.** exp Token Economy

**151.** token*.mp

**152.** (token adj1 economy).mp

**153.** raffle*.mp

**154.** (contingen* adj3 manag*).mp

**155.** (contingent* adj3 contract*)

**156.** lotter*.mp

**157.** coupon*.mp

**158.** voucher*.mp

**159.** gift*.mp

**160.** motivat*.mp

**161.** reinforce*.mp

**162.** punish*.mp

**163.** exp Punishment

**164.** penalt*

**165.** competition*.mp

**166.** contest*.mp

**167.**bonus*

**168.** (contingen* adj3 pay*).mp

**169.** deposit*

**170.** (deposit* adj3 contract*).mp

**171.** disincentive*.mp

**172.** endowment*.mp

**173.** (cash adj3 contingen*).mp

**174.** (pay* adj3 contingen*)

**175.** 131 OR 132 OE 133 OE 134 OR 135 OR 136 OR 137 OR 138 OR 139 OR 140 OR 141 OR 142 OR 143 OR 144 OR 145 OR 146 OR 147 OR 148 OR 149 OR 150 OR 151 OR 152 OR 153 OR 154 OR 155 OR 156 OR 157 OR 158 OR 159 OR 160 OR 161 OR 162 OR 163 OR 164 OR 165 OR 166 OR 167 OR 168 OR 169 OR 170 OR 172 OR 173 OR 174

**176.** randomi?ed controlled trial.pt

**177.** controlled clinical trial.pt.

**178.** randomi?ed.ab.

**179.** placebo.ab.

**180.** drug therapy.fs.

**181.** randomly.ab.

**182.** trial.ab.

**183.**groups.ab

**184.** 176 OR 177 OR 178 OR 179 OR 180 OR 181 OR 182 OR 183

**185.** 130 AND 175 AND 184

**Results limited to Humans and All Adults (18+)**

#

# Text S2. Studies included in review

1. Bloch, M. J., Armstrong, D. S., Dettling, L., Hardy, A., Caterino, K., & Barrie, S. (2006). Partners in lowering cholesterol: Comparison of a multidisciplinary educational program, monetary incentives, or usual care in the treatment of dyslipidemia identified among employees. *Journal of Occupational &* *Environmental Medicine*, *48*, 675-681.
2. Crowley TJ, Macdonald MJ, Walter MI (1995). Behavioral anti-smoking trial in chronic obstructive pulmonary disease patients. *Psychopharmacology* 119, 193–204.
3. Donatelle RJ, Prows SL, Champeau D, Hudson D (2000) Randomised controlled trial using social support and financial incentives for high risk pregnant smokers: Significant Other Support (SOS) program. *Tobacco Control*, 9(Supplement III):iii67–iii69.
4. Donatelle, R. J., S. L. Prows, et al. (2000) Using social support, biochemical feedback, and incentives to motivate smoking cessation during pregnancy: comparison of three intervention trials. American Public Health Association Meeting, Boston MA .
5. Donatelle, R. J. and D. Hudson (2002) Using 5 A's and incentives to promote prenatal smoking cessation. National Conference of Tobacco or Health; 2002 November 19-21; San Francisco, California, USA.
6. Francisco, V. T., A. L. Paine, et al. (1994). "An experimental evaluation of an incentive program to reduce serum cholesterol levels among health fair participants." *Archives of Family Medicine* 3 (3), 246-251.
7. [Galbo, S (2011). Worksite Weight Loss Intervention Utilizing Monetary Incentives and Contingency Management for Overweight and Obese Employees at Risk for Type 2 Diabetes](http://digitalcommons.uconn.edu/cgi/viewcontent.cgi?article=1201&context=gs_theses), University of Connecticut, available here <http://digitalcommons.uconn.edu/cgi/viewcontent.cgi?article=1201&context=gs_theses>

- LahiriI, S. & Faghri, P. D. 2012. Cost-effectiveness of a workplace-based incentivized weight loss program. *Journal of Occupational and Environmental Medicine,* 54**,** 371-377.

1. Gallagher, S. M., P. E. Penn, et al. (2007) A comparison of smoking cessation treatments for persons with schizophrenia and other serious mental illnesses. *Journal of Psychoactive Drugs,* 39, 487-497.
2. Gine, X., D. Karlan, et al. (2010). Put Your Money Where Your Butt Is: A Commitment Contract for Smoking Cessation*. American Economic Journal: Applied Economics* 2(4), 213-235.
3. Glasgow, R. E., J. F. Hollis, et al. (1993). "Results of a year-long incentives-based worksite smoking cessation program." *Addictive Behaviors* 18(4), 455-464.
   - Glasgow, R. E., J. F. Hollis, et al. (1990) Employee and organizational factors associated with participation in an incentive-based worksite smoking cessation program. *Journal of Behavioral Medicine,* 403-418.
   - Glasgow, R. E., J. F. Hollis, et al. (1991) Implementing a year-long worksite-based incentive program for smoking cessation. *American Journal of Health Promotion,* 192-199.
4. Gomel, M., B. Oldenburg, et al. (1993) Work-site cardiovascular risk reduction: a randomised trial of health risk assessment, education, counselling and incentives. *American Journal of Public Health*, 83, 1231-1238.

- Gomel, M. K., B. Oldenburg, et al. (1997). "Composite cardiovascular risk outcomes of a work-site intervention trial." *American Journal of Public Health* 87(4), 673-676.

1. Heil, S. H., S. T. Higgins, et al. (2008) Effects of voucher-based incentives on abstinence from cigarette smoking and fetal growth among pregnant women. *Addiction,* 103(6), 1009-1018.

- Heil, S. H., S. T. Higgins, et al. (2007) Voucher-based incentives for abstinence from cigarette smoking in pregnant and postpartum women. Society for Research on Nicotine and Tobacco 13th Annual Meeting; 2007 Feb 21-24; Austin, Texas 25, Abstract no: PA26-21.
- Higgins, S. T., I. M. Bernstein, et al. (2010) Effects of smoking cessation with voucher-based contingency management on birth outcomes. *Addiction* 105(11) 2023-2030.
- Higgins, S. T., S. H. Heil, et al. (2006) Smoking status in the initial weeks of quitting as a predictor of smoking-cessation outcomes in pregnant women. *Drug and Alcohol Dependence*, 85, 138-141.

1. Hennrikus, D. J., R. W. Jeffery, et al. (2002) RESEARCH AND PRACTICE-The SUCCESS Project: The Effect of Program Format and Incentives on Participation and Cessation in Worksite Smoking Cessation Programs*. American Journal of Public Health*, 92, 274-279.
2. Higgins, S. T., S. H. Heil, et al. (2004). A pilot study on voucher-based incentives to promote abstinence from cigarette smoking during pregnancy and postpartum*. Nicotine & Tobacco Research* 6(6): 1015-1020.

- Higgins, S. T., S. H. Heil, et al. (2006) Smoking status in the initial weeks of quitting as a predictor of smoking-cessation outcomes in pregnant women. *Drug and Alcohol Dependence*, 85, 138-141.
- Higgins, S. T., I. M. Bernstein, et al. (2010) Effects of smoking cessation with voucher-based contingency management on birth outcomes. *Addiction* 105(11) 2023-2030.

1. Higgins et al (unpublished) (reported Higgins 2012).

- Higgins et al (2012). Financial incentives for smoking cessation among pregnant and newly postpartum women *Preventive Medicine* 55 (2012) S33–S40.

1. Hunter, R (2011) Can we *nudge* the population to be more physically active? A randomised controlled trial'. Presentation at UKSB Annual Society Meeting, Stirling, December 2011.
2. Jason, L. A., D. Salina, et al. (1997) A worksite smoking intervention: a 2 year assessment of groups, incentives and self-help. *Health Education Research*, 12, 129-138.

- Jason, L. A., S. D. McMahon, et al. (1995) Assessing a smoking cessation intervention involving groups, incentives, and self-help manuals. *Behavior Therapy,* 26, 393-408.
- McMahon, S. D. and L. A. Jason (2000). Social support in a worksite smoking intervention. A test of theoretical models. *Behavior Modification* 24(2), 184-201.
- McMahon, S. D., L. A. Jason, et al. (1994) Stress, coping, and appraisal in a smoking cessation intervention. *Anxiety, Stress and Coping*, 7, 161-171.
- McMahon Sd, J. L. A. (1998) Stress and coping in smoking cessation: A longitudinal examination. *Anxiety, Stress and Coping*, 11, 327-343.

1. Jeffery, R. W., W. M. Gerber, et al. (1983). Monetary contracts in weight control: effectiveness of group and individual contracts of varying size. *Journal of Consulting & Clinical Psychology* 51(2), 242-248.
   - Jeffery, R. W., W. M. Bjornson-Benson, et al. (1984). "Behavioral treatment of obesity with monetary contracting: two-year follow-up." *Addictive Behaviors* 9(3), 311-313.
2. Jeffery, R. W. and et al. (1984). "Effectiveness of monetary contracts with two repayment schedules of weight reduction in men and women from self-referred and population samples." *Behavior Therapy* 15(3), 273-279.
3. Jeffery, R. W., W. L. Hellerstedt, et al. (1990). "Correspondence programs for smoking cessation and weight control: A comparison of two strategies in the Minnesota Heart Health Program." *Health Psychology* 9(5), 585-598.
4. Jeffery, R. W., R. R. Wing, et al. (1993) Strengthening behavioral interventions for weight loss: a randomized trial of food provision and monetary incentives. *Journal of Consulting and Clinical Psychology,* 61, 1038-1045.
   - Jeffery, R. W. and R. R. Wing (1995). Long-term effects of interventions for weight loss using food provision and monetary incentives.*Journal of Consulting & Clinical Psychology* 63(5), 793-796.
5. Jeffery, R. W., R. R. Wing, et al. (1998) Use of personal trainers and financial incentives to increase exercise in a behavioral weight-loss program. *Journal of Consulting and Clinical Psychology,*  66, 777-783.
6. John et al (2011) Financial Incentives for Extended Weight Loss: A Randomized, Controlled Tria *Journal of General Internal Medicine*. 26(6), 621–626.
7. Klem, M. L. and R. C. Klesges (1988) Competition in a minimal-contact weight-loss program. *Journal of Consulting and Clinical Psychology*, 56, 142-144.
8. Klesges, R. C., R. E. Glasgow, et al. (1987) Competition and relapse prevention training in worksite smoking modification. *Health Education Research*, 2, 5-14.
9. Kramer F, Jeffery R, Snell M, Forster J (1986). Maintenance of successful weight loss over 1 year: effects of financial contracts for weight maintenance or participation in skills training. *Behavior Therapy* 17, 295–301.
10. Long JA et al. (2012). Peer mentoring and financial incentives to improve glucose control in African American veterans: A randomized trial. *Annals of internal medicine,* 156, 416-424.
11. Mahoney MJ. (1974). Self-reward and self-monitoring techniques for weight control. *Behavior Therapy*, 5, 48–57.
12. Norton, S. Richard, et al. (1980) Commitment Contingencies in the Behavioral Treatment of Obesity. Paper presented at the Annual Convention of the Rocky Mountain Psychological Association (50th, Tucson, AZ, April 9-12, 1980).
13. Rand, C. S., M. L. Stitzer, et al. (1989) The effects of contingent payment and frequent workplace monitoring on smoking abstinence. *Addictive Behaviors,* 14, 121-128.
14. Saccone, A. J. and A. C. Israel (1978) Effects of experimenter versus significant other-controlled reinforcement and choice of target behavior on weight loss. *Behavior Therapy*, 9, 271-278.
15. Shoptaw, S., E. Rotheram-Fuller, et al. (2002) Smoking cessation in methadone maintenance*. Addiction*, 97, 1317-1328.
16. Tevyaw, T. O., S. M. Colby, et al. (2009) Contingency management and motivational enhancement: a randomized clinical trial for college student smokers. *Nicotine & tobacco research*, 11, 739-749.
17. Volpp, K. G., L. K. John, et al. (2008) Financial incentive-based approaches for weight loss: a randomized trial. *JAMA*, 300. 2631-2637.
18. Volpp, K. G., A. Gurmankin Levy, et al. (2006). A randomized controlled trial of financial incentives for smoking cessation. *Cancer Epidemiology, Biomarkers & Prevention* 15(1), 12-18.
19. Volpp, K. G., A. B. Troxel, et al. (2009) A randomized, controlled trial of financial incentives for smoking cessation. *The New England Journal of Medicine,* 360, 699-709.
20. Windsor, R. A., J. B. Lowe, et al. (1988). "The effectiveness of a worksite self-help smoking cessation program: A randomized trail." *Journal of Behavioral Medicine* 11(4), 407-421.

- Windsor, R. A. and J. B. Lowe (1989) Behavioral impact and cost analysis of a worksite self-help smoking cessation program. *Progress in Clinical and Biological Research* 231-242.

1. Wing RR, Epstein LH, MarcusM, Shapira B (1981). Strong monetary contingencies for weight loss during treatment and maintenance.*Behavio Therapy* 12, 702–710.
2. Wing RR, Jeffery RW, Pronk N, et al (1996). Effects of a personal trainer and financial incentives on exercise adherence in overweight women in a behavioral weight loss program. *Obesity Research* 4, 457– 62.

# Supplementary Figures

**
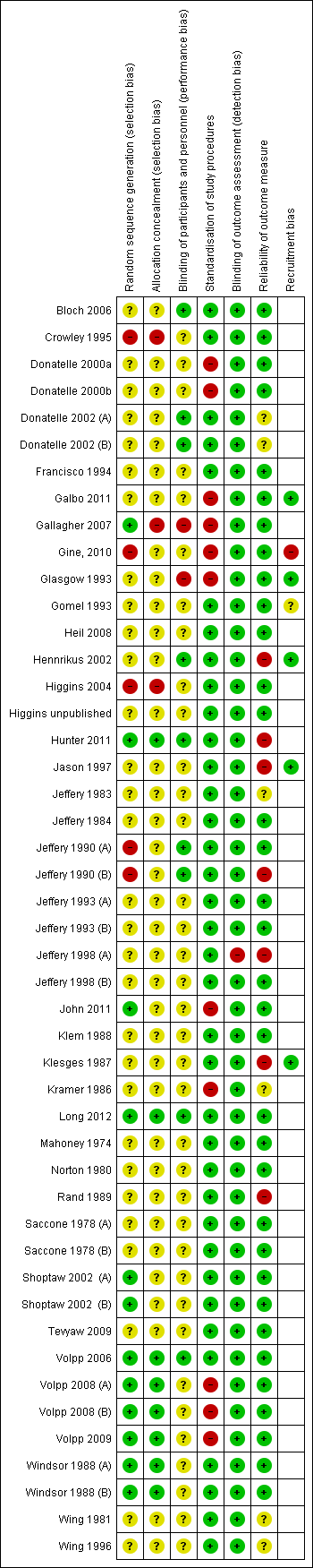
Figure S1. Risk of bias summary: review authors' judgements about each risk of bias item for each included study.**

# Supplementary Tables

**Table S1. Characteristics of included studies**

| **Study;**  **Design** | | | **Country; Setting** | | | **Participants** | | | | **Depriv**  **level** | | **Conditions** | | | **Incentive** | | **Outcomes** | | | **Sustainment of changes** | | | **Notes** | |  |
| --- | --- | --- | --- | --- | --- | --- | --- | --- | --- | --- | --- | --- | --- | --- | --- | --- | --- | --- | --- | --- | --- | --- | --- | --- | --- |
| **Smoking cessation** | | | | | | | | | | | | | | | | | | | | | | | | |  |
| Crowley  1995  RCT | | | USA; COPD clinic | | | 49 smokers with diagnosis of COPD | | | | High | | All participants received a brochure + nicotine gum + CO monitoring over 86 days. They were encouraged to throw cigarettes down toilet. All were given 1 lottery ticket/ day for ’time and effort’.  3 groups:  **1.Exp Group:** Rewarded with lottery tickets for every CO test < 10 ppm.  **2. CSR Group:** rewarded with lottery tickets for each self-report of abstinence since last visit.  **3 Control Group:** each control was paired with an exp participant and received the same reward as exp ’partner’ | | | High value; uncertain | | Mean CO levels and cigarettes smoked per day at 6 months  Validation: expired CO (cut off 10ppm), urinary cotinine, finger pulse oximetry for blood oxygen saturation | | | Assessed. Incentives scheme duration was 65 days and the final assessment was at 6 months | | | CSR group excluded from analyses -it assesses the impact of a variable not evaluated by the review | |  |
| Donatelle 2000a  RCT | | | USA; prenatal clinics of WIC program. | | | 220 pregnant smokers, WIC eligible | | | | High | | 2 groups:  **1. Best Practice As**  (ask, advice, assess, assist, arrange) which included information on the importance of quitting smoking during pregnancy and tailored smoking cessation kit.  **2. Best Practice As+ $50/month voucher** + opportunity to choose supporting partner who also received vouchers. | | | High value; certain | | Smoking cessation at 8 months gestation and 2 months post-partum.  Validation: salivary cotinine /<30ng/ml; salivary thiocyanate monthly /<100mg/ml | | | Not assessed. Incentives removed at final assessment (8 months) | | |  | | |
| Donatelle 2000b  RCT | | | USA; prenatal clinics of WIC program | | | 186 pregnant smokers recruited from prenatal clinics from 8 Oregon WIC program counties | | | High | | | 3 groups:  **1. Best Practice As** (ask, advice, assess, assist, arrange) which included information on importance of quitting smoking during pregnancy and tailored smoking cessation kit.  **2. Best Practice 5 A’s + $25/month voucher** for biochemically confirmed cessation +opportunity to select a partner to support them (supporter did not receive incentives).  3**. Best Practice 5 A’s plus $25/month voucher for biochemically confirmed cessation** + **biochemical feedback of potential harm to baby** + select a partner to support them | | Low value; certain | | | Smoking cessation at 8 months gestation.  Validation: salivary cotinine /<30ng/ml; CO/<5ppm | | | Not assessed. Incentives removed at final assessment (6 months) | | | Best Practice 5 A’s plus $25/month voucher + biochemical feedback of potential harm group excluded from analyses -not an appropriate control | |  |
| Donatelle 2002  RCT | | | USA; prenatal clinics of WIC program | | | 592 pregnant smokers recruited from prenatal clinics participating in WIC program | | | High | | | 3 groups:  **1.** **Best Practice As**  support (ask, advice, assess, assist, arrange)which included information on importance of quitting smoking during pregnancy and tailored smoking cessation kit.  **2.Best Practice 5 A’s plus $25/month voucher** for biochemically confirmed cessation  **3. Best Practice 5 A’s plus $75/month voucher** for biochemically confirmed cessation | | Group 2: Low value; certain  Group 3: High value; certain | | | Smoking cessation at 8 months gestation, 2 months post-partum and 6 months post-partum  Validation: via salivary cotinine /<30ng/ml; salivary thiocyanate monthly /<100mg/ml at | | | Assessed. Incentives removed at 8 months gestation (scheme duration 6 months) and final assessment was at 6-months post-partum | | | **Data provided by author** **upon request**  Study includes two incentivized groups differing in a key potential effect modifier (i.e. value) Figures in the control group were divided by 2 to derive to 2 comparisons, each added to the analyses as if from independent studies. | |  |
| Gallagher 2007  RCT | | | USA; psychiatric facilities | | | 180 mental health patients who smoked. | | | High | | | 3groups:  **1. CR:** Weekly visits on wks (1-4 Phase I), fortnightly on wks 6-12 (Phase II) monthly on wks 16-24 (Phase III). Payments of $20 per abstinent visit in Phase I, $40 in Phase II, $60 in Phase III, and $80 if abstinent at 36wk follow up.  **2.CR+NRT:** As CR Group, + 16-wk course of 21mg NRTpatches, + supporting instructions. **3. Control:** Visits at baseline and wks 20 and 36, +encouraged use of community smoker helpline and self-help information. | | High value; certain | | | Smoking cessation at 20 weeks and 36 weeks.  Validation: Verified by expired CO < 10ppm and by salivary cotinine <15ng/mL. | | | Not assessed. Incentives removed at final assessment (9 months) | | | The CR +NRT group excluded from analyses-not appropriate control | |  |
| Gine, 2010  Cluster-RCT | | | Philippines; Community | | | 2000 members of the general public who smoked, approached by Green bank representatives on the street and asked to participate  Randomization was at the area level | | | High | | | All participants received an information pamphlet on the dangers of smoking and a tip sheet on how to quit.  4.groups:  **1. CARESw/ deposit:** financial commitment in form of savings balances and a non-financial commitment to be visited by a deposit collector (social pressure).Smokers encouraged to deposit their cigarette money every wk for 6 months, which was forfeited if failed smoking test. Participants received 30 pesos for taking 12 month test  **2. CARES w/out deposit :** As above but without the deposit element (group dropped)  **3. Cue cards**  **4. Control:** Participants offered 30 pesos for taking 6 month test + 30 for 12 month test. | | High value; certain | | | Smoking cessation at 6 months and 12 months  Validation: urine cotinine equal to zero | | | Assessed. Incentives scheme duration was 6 months and final assessment was at 12 months | | | The authors dropped the CARES w/out deposit group due to low uptake and the Cue cards is excluded from analyses because an appropriate control | |  |
| Glasgow 1993  Cluster-RCT | | | USA; workplace | | | 18 worksites recruited from pre-specified areas including 1097 employee smokers | | | Other | | | 2 groups:  **1. Incentive:** Participants were eligible to receive monthly incentives ($120) for a year and if abstinent entered into monthly worksite lottery and annual sweepstake. Personnel offered support and encouragement but no quitting advice and support.  **2. Non-Incentive:** Participants completed baseline and follow up surveys at 1 year and 2 years follow-up | | High value; certain and uncertain | | | Smoking cessation at 1 year and 2 years.  Validation: CO below 9ppm and salivary cotinite below 15ng/ml | | | Assessed. Incentives scheme duration was 1 year and last assessment was at 2 years | | |  | |  |
| Gomel 1993  Cluster-RCT | | | Australia; workplace | | | 28 ambulance stations involving 219 smokers. | | | Other | | | 4 groups:  **1. HRA:** risk factor profile feedback  **2. RFE**: as above +advice, brochure, videos  **3. BC:** as group 2 +individual counselling+ life-style change manual  **4. BCI:** as group 3 +incentives, i.e. 2 lottery draws for A$40 over 10wk period, + 5 draw tickets for 1wk cessation; At 3m A$40 voucher for achieved targets. Station achieving highest % of participants meeting 6m goals won A$1000. | | High value; certain and uncertain | | | Continuous smoking cessation; Change in BMI; Change in % of bay fat; Mean cholesterol changes; Change in aerobic capacity at 6 month and 12 months.  Validation with blood tests (for smoking cessation blood cotinine was calculated with cut of 100), weight and height measurements; skin fold thickness measurements with clippers; Max oxygen consumption | | | Assessed. Incentives scheme duration was 6 months and last assessment was at 12 months | | | Full data available only for smoking cessation. Data related to other outcomes were excluded from the analysis.  HRA and RFE groups excluded from analyses-not appropriate controls | |  |
| Heil 2008  RCT | | | USA; prenatal clinic of WIC program | | | 82 pregnant smokers recruited from one of four local large group obstetric practices and the WIC program | | | High | | | All participants chose quit date, and reported daily to clinic for CO monitoring for 5 days, then urine cotinine monitoring twice wkly for 7 wks, wkly for 4 wks, and then every 2 weeks for remainder of pregnancy. Post partum monitoring increased to once/wk for initial 4 wks, and then biwkly for next 8 wks, with abstinence monitoring ending at end of wk 12.Vouchers escalating in value given for smoking cessation. Non-cessation reset vouchers back the original value but 2 consecutive negative tests restored value to pre-reset value. Participants also received routine advice from clinic.   2 groups:  **1.Contingent voucher group:** participants received vouchers for cessation beginning at US$6.25 and escalated by US$1.25 to a max of US$45.00. +ve test results reset voucher back to original value  **2. Non-contingent voucher group:** Participants received voucher independent of smoking status. US$15.00 per antenatal visit and US$20.00 per postpartum visit | | High value; certain | | | Smoking cessation at end of pregnancy, 12 weeks post-partum and 24 weeks post-partum.  Validation: confirmation of abstinence was with CO /< 6ppm for the first 5 days and then with  urine-cotinine levels /<80 ng/ml | | | Assessed. Incentives removed at 12 weeks post-partum (scheme duration 8 months) and last assessment was at 24 weeks post-partum | | |  | |  |
| Hennrikus 2002  Cluster -RCT | | | USA; workplace | | | 2402 smokers recruited from 24 worksites. | | | Other | | | 3 groups:  **1. Group:** 13 group sessions over 2months  **2. Phone:** sent printed materials+ 3-6 telephone counselling sessions **3. Choice:** free choice between group or phone program.  Half sites in each intervention were offered direct incentives for participation and quitting: Quitters at 1month won $20 and entered lottery for grand prize drawn about every 6months | | High; uncertain | | | Smoking cessation at 12 months and 24 months.  Validation: self-report, countersigned by friend or family member for monthly abstinence. Grand draw prize winners + 24 month random sample of quitters tested for salivary cotinine | | | Assessed. Incentives scheme duration was 18 months and last assessment was at 24 months | | | Two levels of incentives were crossed with 3 program formats: groups sessions, telephone sessions or a choice, leading to 6 possible groups. For the analyses, groups of different format were combined to assess the effect of incentive vs. no incentive | |  |
| Higgins 2004  RCT | | | USA; prenatal clinics | | | 58 pregnant smokers recruited from one of three large group local obstetric practices or one single-practitioner obstetric practice | | | High | | | Same as Heil 2008 | | High value; certain | | | Smoking cessation at 36 weeks gestation, 12 weeks post-partum and 24 weeks post-partum  Validation: confirmation of abstinence was with CO /< 6ppm for the first 5 days and then with  urine-cotinine levels /<80 ng/ml | | | Assessed, Incentives were removed at 12 weeks post-partum (duration 9.5 months) and last assessment was at 24 weeks post-partum | | |  | |  |
| Higgins unpublished (mentioned in Higgins 2012)  RCT | | | USA; prenatal clinics | | | 48 pregnant smokers recruited from local obstetric practices and WIC program. | | | High | | | Same as Heil 2008 | | High value; certain | | | Smoking cessation at 36 weeks gestation, 12 weeks post-partum and 24 weeks post-partum.  Validation: confirmation of abstinence was with CO /< 6ppm for the first 5 days and then with  urine-cotinine levels /<80 ng/ml | | | Assessed, Incentives were removed at 12 weeks post-partum (duration 9.5 months) and last assessment was at 24 weeks post-partum | | |  | |  |
| Jason 1997  Cluster-RCT | | | USA; workplace | | | 844 employee smokers recruited from 63 worksites | | | Other | | | 3 groups:  **1. Self-help group:** 5-day cessation TV program ’Smoke-free in the 90s’+ 8-page newspaper supplement, self-help  **2. Incentives group**: as group 1+ $1/day for each day abstinent up to 6months (maximum $175) **3. Support group:** as group 2 +group meetings twice/week for first 3wks, +14 ’booster’ meetings over 6months; program included ‘buddy’ system, and tips in booster sessions on living with a smoker, weight control, exercise and stress management | | Low value; certain | | | Smoking cessation at 6 months, 12 months, 18 months and 24 months.  Validation: confirmation of abstinence was with CO < 9ppm and for the 6 month assessment saliva cotinine (cut of point not mentioned) was also used | | | Assessed. Incentive scheme duration was 6 months and last assessment was at 2 years. | | | The Support group assessed the effect of group support and cognitive coping skills on smoking cessation and is excluded from the analyses | |  |
| Jeffery 1990 (A)  RCT | | | USA; Community | | | 1304 participants from 31400 households recruited via direct mail. | | | Other | | | 2 groups:  **1. Newsletter:** Participants were posted self-help manuals for smoking cessation and 6 newsletters (one a month) providing advice on behavior change strategies. They were also requested to pay $5 registration fee  **2. Newsletter and incentive:** As above but participants did not pay registration fee but were requested to deposit $60, 1/6 of which would be refunded each month they were abstinent. Full refunds given to those achieving at least 4 month cumulative abstinence or abstinence in last month of program. | | Low value; certain | | | Smoking cessation at 6 months; Validation: Participants reporting being abstinent at the end of the study were required to come in for validation with salivary cotinine less than 11 ng/ml | | | Not assessed. Incentives removed at final assessment (6 months) | | |  | |  |
| Klesges 1987  Cluster-RCT | | | USA; workplace | | | 136 employees from 8 worksites recruited via posters, pamphlets and in-house memos announcing a smoking program at their worksite; | | | Other | | | 3 groups:  **1. Basic Program** 6 wkly CBT group sessions, aimed at brand-switching+reduction, aiming for final quitting or reduced % of each cig smoked. Also info on maintenance and relapse prevention **2. Competition:** As 1, + within-site team competitions. Wkly feedback on team performance, smoking ’barometer’, prizes for completing treatment (~ $5 per team member), for team with highest number of quitters at end (~ $10 per member), and for highest abstinence at 6m follow up (~ $15 per member). **3. Relapse prevention:** After 6 wks intervention worksites were randomized to relapse prevention or no relapse prevention resulting in 4 groups to be assessed at the 6 month follow-up  As 1, +/- Comp, +/- 1- or 2-monthly meetings to discuss, role-play, quit again, develop RP skills. | | Low value; certain | | | Smoking cessation at 6 months;  Validation: CO < 10 ppm and SCN at baseline. CO preferred to SCN at 6m follow up. | | | Not assessed. Incentives removed at final assessment (6 months) | | | Relapse prevention vs. no relapse prevention groups were collapsed for the analyses to assess the effect of incentive vs. no incentive at 6 months | |  |
| Rand 1989  RCT | | | USA; workplace | | | 51 smokers employed at a medical center recruited with poster adds and word of mouth | | | High | | | 3 groups:  **1. Contingent payment/frequent monitoring:** Participants attended initial 15min lecture on strategies for smoking cessation+were given a booklet. Were told to stop smoking the following Sunday in preparation for Monday morning start of monitored abstinence period (5 days) when participants were visited at worksites for collection of breath samples. Were also required to visit study site to give afternoon sample+were visited at home for collection of evening samples. They received $25 for completion of this phase. Successful abstainers were visited at worksites twice/for breath samples+were paid $4 for each CO value less than 11ppm.  **2. Non-contingent payment/frequent monitoring:** As above but participants paid $4 regardless of CO values  **3. Non/contingent payment infrequent monitoring:** As above but participants were monitored only at 6 months | | Low value; certain | | | Smoking cessation at 6 months;  validation: CO reading equal or below 11ppm | | | Not assessed. Incentives removed at final assessment (6 months) | | | Non contingent payment/frequent monitoring group was the most appropriate comparison. Therefore the non-contingent/infrequent monitoring group was excluded from the analyses | |  |
| Shoptaw 2002  RCT | | | USA; Methadone maintenance clinic | | | 175 drug addict smokers were identified through flyers and counsellor nominations | | | High | | | 4 groups:  **1. Patch:** For duration of the 12 weeks prog participants received two or three nicotine patches with max of 84 patches. Participants provided breath +urine samples 3 times/wk  **2. Patch & CM:** As above+participants were offered vouchers for smoking cessation, escalating in value with consecutive abstinent CO samples and reset in case of failure.  **3. Patch & RP:**As 1+ participants received relapse prevention counselling: psycoeducational techniques to enhance coping with smoking cessation +behavioral skills to instil smoking abstinence+mood management  **4. Patch& CM &RP:** Participants received patch+ offered vouchers+ underwent relapse prevention | | High value; certain | | | Smoking cessation at 6 months and 2 months;  Validation:Self-report, CO level at or below 8ppm and urine cotinine below 30ng/ml | | | Assessed. Incentive scheme duration was 12 weeks and final assessment was at 12 months | | | Comparisons of interest: i) Patch vs. Cm ii) Patch & RP vs. Parch & RP & CM. Each entered into analyses as if from independent studies (dummy record generated) | |  |
| Tevyaw 2009  RCT | | | USA; Universities | | | 110 students smokers recruited from colleges and universities in a north-eastern U.S. state | | | Other | | | Four groups:  **1.MET + CM:** Participants underwent motivational therapy+had 2 daily CO breath samples collected+received $5 for samples regardless of smoking status . During wk 1 they earned $1 for CO reduction 25%-49%; $2 for reduction 50%-74% and $3 for 75% reduction or greater. During wk 2 reinforcement given for abstinence in an escalating schedule beginning at $3 and increasing by $0.5 for each consecutive non-smoking sample+ received bonus of $1 for 2 consecutive non abstinent samples. Failure reset value to $3 which returned to to original value after 4 consecutive abstinent samples.  **2. MET + NR(no reinformcement):** Participants received motivational enhancement+had 2 daily CO breath samples collected+received $5 for samples regardless of smoking+bonus of $10 for attending at least 80% of the CO readiness for each wee. Total of $240 could be earned.  **3. REL (muscle relaxation) +CM:** As MET & CM but instead of motivation therapy participants received muscle relaxation therapy  **4. REL + NR:** Participants received muscle relaxation+gave 2 daily CO breath samples+received $5 for samples regardless of status+bonus of $10 for attending at least 80% of the CO readiness for each wee. Total of $240 could be earned. | | Low value; certain | | | Smoking cessation at 6 months;  Validation: CO reading /<5ppm and saliva cotinine <15ng/mL | | | Assessed. Incentive scheme duration was 3 weeks and final assessment was at 3 months | | | The 2 MET groups and the 2 REL were collapsed for the analysis to focus on CM vs. NR in line with the review by Cahill & Perera (2011) | |  |
| Volpp 2006  RCT | | | USA; medical centrer | | | 176 smokers recruited from the waiting room of an outpatients’ clinic; | | | High | | | 2 groups:  **1. Incentives:** Participants received invitation to enrol in smoking cessation prog+offered $20 to attend each of 5 sessions (total of $100) + $100 for self-reported quitting at 30 days+nicotine patches+$20 for adhering to validation procedures  **2. No incentives:** Participants received invitation to enrol to smoking cessation prog. All enrolled received free nicotine patches+$20 for adhering to validation procedures | | Low value; certain | | | Enrolment at smoking cessation program; attendance and completion of smoking cessation program; 7-day point prevalence smoking cessation at 6 months following program completion;  Validation: urine cotinine (<500 ng/mL) | | | Assessed. Incentive scheme duration was 30 days and last assessment was at 6 months | | |  | |  |
| Volpp 2009  RCT | | | USA; workplace | | | 878 smokers employees of a big company recruited through survey | | | Other | | | All participants received $20/ telephone interview at baseline and at 3months follow up+ $25/ confirmatory sample returned.  2 groups:  **1. Incentive:** Participants received $100 for completion of smoking-cessation prog; $250 for smoking cessation confirmed with cotinine test within 6 months after study enrolment; $400 for continued abstinence 6 months after initial cessation (9 or 12 months after enrolment)  **2. Control:** Participants in this group received information about smoking cessation programs without being offered financial incentives. | | High value; certain | | | Prolonged abstinence at 9 or 12m. Those not abstinent at 3m were retested at 6m, and followed from then if abstinent. All abstinent at both follow ups were assessed again 6m later, i.e. at 15 or 18m.  Validation: Cotinine by saliva 15 ng per ml or urine cotinine level of 2ng per ml | | | Assessed. Incentive scheme duration was 9 or 12 months and last assessment was at 15 or 18 months | | | 9-12m endpoint used in 6m time-point, and 15-18m endpoint in 12m time-point, in line with Cahill & Perera’s (2011) analyses. | |  |
| Windsor 1988  RCT | | | USA; workplace | | | 378 employees smokers informed about the smoking cessation program through announcement in company newspaper | | | Other | | | 4 groups:  **1.Control/self-help manual onl**y: Participants received self-help manual  **2. Self-help manual &social support/skills training:** As 1+ received cessation skills training (diary, deep breathing), contract to quit+quit smoking ’buddy’ (with buddy education).  **3.Self- help manual& incentives:** As 1+ received an incentive 6 wks following cessation and another at end of 6 month cessation (total $50)  **4.Self- help manual, social support/skills training&incentives:** As groups 1,2 and 3 combined | | Low value; certain | | | Smoking cessation at 6 months and 12 months;  Validation: SCN with a cut off of /<100mg/ml | | | Assessed. Incentive scheme duration was 6 months and last assessment was at 1 year | | | Comparisons of interest are i)Group 1 vs 3 ii) Group 2 vs 4. Each comparison entered into analyses as if from independent studies (dummy record generated) | |  |
| **Indicators of healthier eating and/or physical activity** | | | | | | | | | | | | | | | | | | | | | | | | |  |
| Bloch 2006  RCT | | USA; workplace | | | 171 adults attending an annual health screening program found to have high cholesterol. | | | Other | | All participants received on-line educational materials  3groups:  **Group 1** received $100 for reduction of LDL-C by 15% within 6 months  **Group 2** received multi-disciplinary educational program (series of live classes and phone support delivered by a nurse educator)  **Group 3** received no further intervention. | | | | | Low value; certain | | Mean % change in LDL-C at 6 months compared to baseline; number of individuals in each group reaching target reduction of 15% in LDL-C at 6 months.  Validation: fasting blood test | | Not assessed. Incentives removed at final assessment (6 months) | | | Group 2 excluded from analyses -not an appropriate comparison group | | |  |
| Francisco 1994  RCT | | USA; workplace | | | 63 employees with elevated cholesterol, recruited from a health fair. | | | Other | | All participants received health-related information at health fair  2 groups:  **1.Experimental group:** offered $100 to reduce serum cholesterol by 20% or below 5.17mmoI.L (200mg/dL)  **2. Control group:** not offered incentive to reduce serum cholesterol | | | | | Low value; uncertain | | Mean change in serum cholesterol level at 6 months after pre-test;  Validation:Non-fasting blood test | | Not assessed. Incentives removed at final assessment (6 months) | | | Study excluded from analyses: outcome dispersion reported in the form of ranges, which are unstable for the estimation of standard errors (needed for the analyses). | | |  |
| Galbo 2011  Cluster-RCT | | USA; workplace | | | 73 overweight or obese employees working at 4 nursing homes | | | Other | | 2 groups:  **2.Control:** Participants received booklet and had 1hr consultation with dietician and/or Health Educator-set weight loss goals (16lb or 24lb in 16 weeks depending on initial BMI). They were weighted at 16 weeks and 28 weeks.  **2. Incentivized group:** Participants received above care + incentives for losing 11-14lb + could deposit money which could be earned back and matched if goals were met. | | | | | High value; certain | | At 16 weeks: % participants losing weight  At 28 weeks: Mean weight loss; % of participants showing improvement in BMI; **% participants losing weight** **(included in analyses)**;% of participants losing weight/maintaining weight loss | | Not assessed. Incentives removed at final assessment (6 months) | | |  | | |  |
| Jeffery 1983  RCT | | USA; Community | | | 89 overweight adults recruited from a population sample of men surveyed but found ineligible for a different trial | | | Other | | All participants participated in 16 wk behavioral weight-reduction/education program. Weight loss goals were 30lb. Participants weighed wkly.  6 groups:  **1.Group_$30:** Participants deposited $30 and were reimbursed based on average weight-loss of their groups  **2.Group_$150:** As group 1 but participants deposited $150 **3.Group_$300:**As groups 1&2 but participants deposited $300 **4.Individual_$30:** As group1 but participants reimbursed based on own weight-loss at rate of $1/lb up to a max weight loss of 2lb/wk  **5.Individual_$150:** As group 2 but participants were reimbursed based on own weight-loss at a rate of $5/lb up to a max weight loss of 2lb/ week.  **6.Individual_$300:** As group 3 but participants reimbursed based on own weight-loss at a rate of $10/lb up to a max weight loss of 2lb/ week | | | | | Low value; certain | | Weight change at 6, 12 and 24 months | | Assessed. Incentive scheme duration was 16 weeks and last assessment was at 1 year | | | Study excluded from analysis: the two incentivized groups did not differ on any key variables (classification of value and/or certainty of attainment) and there was no control group to which a possible combination of the two could be compared. | | |  |
| Jeffery 1984  RCT | | USA; Community | | | 115 adults half of which were recruited from a population sample of a previous study by Jeffery et al 1983 and the other half through newspaper advertisement. | | | Other | | All participants took part in 16 wkly group meeting covering nutritional, exercise and behavioral principles. A $150 deposit was used to construct weight loss contracts.  3 groups:  **1. Control:** deposit was refunded at first session  **2. Contracts w/ constant refunds:** Participants either received $30 for each 5lb increment of average group weight loss (weight loss goal was 20l for women and 30lb for men)  **3. Contract with increasing refunds:** participants received funds for successive 5lb increments of $5, £10, $20, $40 and $75. Individuals weight loss goals were 20lb for women and 30lb for men | | | | | Low value; certain | | Mean change in body weight at 1 year | | Assessed. Incentive scheme duration was 16 weeks and last assessment was at 1 year | | | The two financial incentive groups differed in a variable not assessed by the review and groups were collapsed for the analyses | | |  |
| Jeffery 1990 (B)  RCT | | USA; Community | | | 142 individuals recruited from 31400 households recruited via direct mail. | | | Other | | 2 groups:  **1. Newsletter:** Participants were posted self-help manuals for weight-loss and 6 newsletters (one a month) providing advice on behavior change strategies. They were also requested to pay $5 registration fee and set a weight-loss goal of max 4lb/month (24lb in 6 months).  **2. Newsletter and incentive:** As above but no registration fee requested. But participants deposited $60 to be refunded proportionate to their weight loss | | | | | Low value; certain | | Mean weight loss at 6 months;  Validation: Weight loss was confirmed via self report and a random sample was weighed | | Not assessed. Incentives removed at final assessment (6 months) | | | Dummy record. Jeffery 1990 (A) and (B) are part of one study report reporting two incentive schemes; one for smoking cessation and one for weight loss | | |  |
| Jeffery 1993  RCT | | USA; Community | | | 202 overweight participants recruited from two urban communities through newspaper add, radio announcement and mailed invitation. | | | Other | | five groups:  **1.Control:** no intervention  **2.SBT:** Participants provided with a standard behavioral treatment: counselling in groups meeting wkly for first 20 wks and once a month thereafter including wkly weigh-ins and individualised caloric daily goals and set weight loss goals (14 18 or 23 kg). Participants asked to record caloric intake in daily food record for first 20 wks and for 1 week each month thereafter. Exercise program also prescribed  **3.SBT + food provision:** Same SBT but participants also received packaged meals for 5 breakfasts and five dinners each wk and meal plan  **4.SBT + incentives:** As SBT but participants also received cash payment each wk based on weight loss. Max payment $25/wk ;min payment $2.5 if weight maintained. Weight-losses of 50% of goal paid with $12.50/wk  **5. SBT+ food provision+incentives:** As SBT group, as well as packaged meals and incentives | | | | | High value; certain | | Change in BMI; Change in weight; Changes in total calories consumed per day;% of calories from fat at 6 months; 12 months, 18 months and 30 months; Estimated energy expenditure from exercise, last week of 18 months | | Assessed. Incentive scheme duration was 18 months and last assessment was at 30 months | | | Control group is excluded from analysis because it is not the most appropriate comparison for assessing the impact of incentives.  Comparisons of interest are between: i) SBT vs. SBT+I ii) SBT+food provision vs. SBT+food provision +I. Comparisons added to analyses as if from independent studies (dummy record generated) | | |  |
| Jeffery 1998  RCT | | USA; community | | | 196 overweight participants recruited by media advertisement from 2 urban communities | | | Other | | five groups:  **1. SB:** Participants received behavioral intervention program for 18 months (group counselling, met wkly for 24 weeks and monthly thereafter). Sessions included weigh-ins and participants were given caloric goals and menus. They were asked to record their daily caloric and fat intake and instructed to exercise and were taught behavioral techniques.  **2. SBT+ supervised walks:** As SB but participants also had 3 supervised walks/wk.  **3. SBT+ supervised walks+ incentives**: As group 1 and 2 but participants received financial incentives based on number of sessions walked at end of each month, increasing in value with cumulative attendance.  **4.SBT+ supervised walls w/ personal trainers:** As group 2 but personal trainer assigned to work with 3-4 participants,  **5. SBT+SW+PT+I:** Received the treatment delivered to all groups | | | | | High value; certain | | Mean change in body weight and exercise behavior at 6 month and 18 months:  Validation: body weight measured with scales; exercise behavior measured with Paffernbarger Physical Activity Questionnaire and number of walk session attended | | Not assessed. Incentives removed at final assessment (18 months) | | | The SB group is excluded from analyses because it is an inappropriate control.  Comparisons of interest: i) SBT plus supervised walks vs. SBT with supervised walks plus incentives ii) SBT with supervised walls with personal trainers vs. SBT+SW+PT+I  Comparisons added to analyses as if from independent studies (dummy record generated) | | |  |
| John 2011  RCT | | USA; community | | | 66 overweight participants identified through the Philadelphia Veterans Medical Center patient database | | | Other | | 3 groups:  **1. Control:** Participants underwent a 1hr consultation with dietician at enrolment, in which strategies for weight loss were discussed, goal-setting and monthly weigh-ins. They were given weight loss target of 24lb for first 24 wks and could then chose goal for wks 24-32. Also given a scale to monitor weight at home and received $20 for returning to clinic to be weighed  **2. Deposit contract- maintenance:** At the beginning of the program participants had a 1h consultation with dietician. Weight loss goal set at 24lb in 24 wks. At beginning of each month they deposited $0-$3/day of their funds. During the month they could accumulate rewards if each day they reported a weight at or below target weight-loss (their own deposit plus a 1:1 match) which they would receive if they weighed at or below target weight loss at the end of month (4 pounds). After 24 wks they received incentives for maintaining weight loss. Participants also encouraged to have daily weigh-ins and received daily text msgs with feedback on progress and earnings.  **3. Deposit contract – opportunity for continued weight-loss:** As above but after 24 weeks people told that during the next phase they had opportunity to continue losing weight and earning incentives (they could lose 24 lb in 32 wks) | | | | | High value; certain | | Mean weight loss; proportion of individuals achieving target weight loss at 32 weeks from start of program and 36 weeks from end of program; participants achieving weight loss equivalent to 5% of initial body weight **(latter data included in analysis and given by authors upon request)**    Validation: weight ins | | Assessed, Incentive scheme duration was 32 weeks and final assessment was at 17 months | | | As the two incentive conditions differed in aspects other than the incentives offered they were collapsed for the analyses | | |  |
| Klem 1988  RCT | | USA; commnuity | | | 59 overweight participants | | | Not known | | 3 groups:  **1. Bibliography:** Participants given a self-help weight loss manual. Each wk for 12 wks they were weighed.  **2. Competition:** Participants were assigned to two teams with cash prizes awarded to the team a) with highest participant rates ($5 per member) b) the highest % weight loss at 12 wks ($10 per member) and c) the best maintenance of weight loss at the 3 month follow-up ($20 per member)  **3. Bibliography + competition:** As group 1 and 2 | | | | | Low value; certain | | Weight loss at 6 months and 9 months ;  Validation: weight ins | | Assessed. Incentive scheme duration was 6 months and last assessment was at 9 months | | | With Bibliography as the control the Bibliography+Competition group is a more appropriate comparison to estimate the effects of incentives. Therefore the Competition group was excluded from the analyses | | |  |
| Kramer 1986  RCT | | USA; Community | | | 139 overweight participants recruited through newspaper advertisement, worksites and ineligibles from other studies | | | Other | | All participants deposited $120  3 groups:  **1. Incentives for weight maintenance:** Participants took part in program with non-specific format. Monthly sessions consisted of discussions about weight maintenance and related problems. Deposit was lost for each session not attended + withheld (but returned in the end) if they weighed more than previous sessions  **2. Incentives for participation in skills training:** Participants received parts of deposit for attending monthly group meetings which provided diet and physical activity information and skills training for maintaining weight loss  **3. No treatment:** No intervention. $100 from deposit refunded immediately. Participants sent reminder letter at 6 months and followed at  1 year when they received the reminder $20 | | | | | Low value; certain | | Change in weight; % of individuals maintaining lost weight at 1 year;  Validation: weigh ins | | Not assessed. Incentives removed at final assessment (12 months) | | | The skills training group is excluded from analyses because it is least comparable to the incentives for weight maintenance group | | |  |
| Long 2012  RCT | | USA; medical center | | | 118 African American participants aged 50 to 70 with persistently low diabetes control, treated at the Philadelphia VA  Medical Center | | | Other | | All patients were called the day after enrolment and notified of their starting HbA1c level as well as the American Diabetes Association  and VA recommendations about HbA1c target levels. Thet were paid $25 for returning 6 months later for the follow-up.  3 groups:  **1.** **Usual care:** received no further intervention  **Peer mentoring:** Patients matched to a peer mentor (African American patients whose glucose control had previously been poor but was currently good) within 1 to 3 wks.  **3. Financial incentives:** participants offered $100 at 6 months for 1% decrease HbA1c and $200 for 2% decrease. | | | | | Low value; certain | | Change in HbA1c levels at 6 months.  Validation: blood test | | Not assessed. Incentives removed at final assessment (6 months) | | | Peer mentoring groups is excluded from analyses because as it is not an appropriate control | | |  |
| Mahoney 1974  RCT | | USA; community | | | 49 overweight participants recruited through newspaper advertisement | | | Not known | | Four groups:  **1.Self-reward for weight loss:** Participants deposited $35 and were fined $5 for missing group meetings. They received pamphlets describing stimulus control strategies for alteration of eating habits+weight charts & eating habits booklets for daily self-monitoring. During first 2 wks they recorded  daily weight and eating habits +attended weight ins.  During next 6 wks they received weight loss & habit improvement goals + were instructed to award themselves portions of their deposit for attainment of wkly goasl  **2.Self-reward for habit improvement:** As group 1 but participants were instructed to award themselves portions of their deposit for attainment of their weekly habit improvement goal  **3. Self-monitoring:** As group 1 but  during the subsequent 6 wks participants continued to monitor themselves and received weight loss and habit improvement goals after each weigh in.  **6. Delayed control** | | | | | Low value; certain | | Reduction quotient (Number of pounds lost divided by number of pounds overweight) at year 1 | | Assessed. Incentive scheme duration was 8 weeks and final assessment at 1 year | | | Study excluded from the analyses: data relating to assessments at a minimum of 6-months from baseline were not reported.  . | | |  |
| Norton 1980  RCT | | USA; community | | | 26 overweight participants recruited from a community of northern Utah | | | Not known | | Three groups:  **1. Commitment for study completion and behavior change:** Participants took part in a 10 wk prog. 2 first wks were for self-monitoring and next 8 were for weight loss. Were weighed twice/ week +attended wkly group meeting where they received social reinforcement and education in stimulus control of eating behavior. Treatment included self-monitoring of eating and exercise and related goal setting.   Participants also made a $15 deposit returned for completing study & meeting goals.  **2.Commitment for study completion:** As group 1 but participants deposited $15 retuned if they completed the study  **3. No commitment** | | | | | Low value; certain | | Behavior changed in terms of eating and exercising; weight loss at 8 months;  Validation: Behavior change measured by self report; weight loss measures at weight ins | | Assessed, Incentive scheme duration was 10weeks and last assessment was at 8 months | | | Study is excluded from the analyses: data relating to assessments at a minimum of 6-months from baseline were not reported. | | |  |
| Saccone 1978  RCT | | USA; community | | | 49 overweight participants recruited | | | Other | | All participants except for those in the No treatment group received the basic stimulus control package  7 groups:  **1.No treatment**  **2.Program only – monitoring weight:** Participants told to monitor their weight daily  **3.Program only – monitoring eating:** Participants told to monitor their eating behavior daily  **4.Program w/ reinforcement by therapist for weight loss:** participants told to monitor weight daily+ were weighed by therapist wkl.+deposited $30+received $1.50/lb lost up to $5 for last 6 wks  **4.Program w/ reinforcement by therapist for eating behavior change:** participants told to monitor eating behavior daily at dinner meal with 9-point check-list+deposited $30+ received money for appropriate behavior: 0-23 points no reinforcement; 24-33 point $1.75; 34-43 points $3.50, 44-53 points $4.50 & 54-63 point $5  **5.Program w/ reinforcement by sig other  for weight loss:** As 4 but a significant other provided reinforcement  **6. Program w/ reinforcement by sig other for eating behavior change:** As 5 but a significant other provided reinforcement | | | | | Low value; certain | | Change in weight at 1 year;  Validation: weight- ins | | Assessed. Incentive scheme duration was 9 weeks and final assessment was at 1 year. | | | Groups 4 and 6 were combined for the analyses and compared to 2    Groups 5 and 7 are combined for the analyses and compared to 3  The no treatment control as it is not an appropriate comparison to assess the effect of incentives and is excluded from the analyses  The two comparisons of interested entered as if from independent studies (Dummy record generated) | | |  |
| Volpp 2008  RCT | | USA; Health center | | | 57 overweight participants recruited through mailings | | | Other | | All participants received 1h consultation with dietician+were encouraged to have daily weigh-ins+received daily text messages giving feedback on progress and earnings. All participants received $20 for attending weight-ins.  3 groups:  **1. Deposit contract:** At beginning of each month participants contributed $0-$3/day of their funds to a deposit contract. During the month they could accumulate rewards if each day they reported a weight at or below their target weight-loss (their own deposit plus a 1:1 match+a $3 fixed payment) which they received  if they weighed at or below their target weight loss at the end of month (4 pounds). Weigh loss goal was 16 pounds in 16 wks.  **2. Lottery:** Participants were eligible for daily lottery with expected value of $3/day if they reported a weight at or below their weight loss goal. Goal was 16 pounds in 16 weeks  **3. Control:** Participants participated in weight monitoring prog involving monthly weigh-ins. | | | | | Group 1: High value; certain  Group 2: High value; uncertain | | Weight-loss after 7 months;  Proportion of participants achieving 5% weight-loss (data given by author)  Validation: weigh ins | | Assessed. Incentive scheme duration was 16 weeks and last assessment was at 7 months | | | Study includes two incentivized groups differing in a key potential effect modifier (i.e. attainment certainty) Figures in the control group were divided by 2 to derive to 2 comparisons, which were added to the analysis as if from independent studies. | | |  |
| Wing 1981  RCT | | USA; Community | | | 40 overweight participants recruited through advertisement placed in local newspapers | | | Not known | | Participants participated in 9 wkly +7 monthly meetings where they were weighed+participated in lecture-discussion of behavioral strategies for weight control+ were given daily caloric goal to produce 2lb/week weight- loss. Charts of eating&exercise were collected from participants wkly.  2 groups:  **1. Payments for weight loss:** Participants brought 15 cheques for $15 to first meeting. One cheque was either returned or forfeited at each meeting. Cheques were returned at wkly intervals on an overall rate of weight loss of  2lb/wk. If cheques were forfeited 2 consecutive wks goals were adjusted.  During maintenance phase (after first 8 weeks) cheques were returned for attendance.  **2. Payments for attendance:** Participants brought 15 cheques for $15 to the first meeting. One cheque was either returned or forfeited at each meeting. The first 8 cheques were returned at wkly intervals for attendance and the last 7 for 2lb/week weight loss. | | | | | Low value; certain | | Weight loss at 13 months | | Assessed. Incentive scheme duration was 9 weeks and last assessment was at 13 months | | | Study excluded from analyses: a crossover method was employed in the delivery of incentives and there was no control group to which a possible combination of the two crossover treatment groups could be compared. | | |  |
| **Physical activity** | | | | | | | | | | | | | | | | | | | | | | | | |  |
| Hunter 2011  RCT | | | Northern Ireland; workplace | | | 406 participants were recruited from Northern Ireland’s main government offices | | Other | | 2 groups:  **1. Financial incentive:** Participants used Physical Activity Loyalty card to self-monitor physical activity levels. Mins of physical activity were converted to points, redeemed for rewards at week 6 and week 12.  Participants received feedback via the PAL scheme website on minss of physical activity calories burned and distance covered.  **2. No financial incentive:** as above but participants unable to collect ‘points’ or earn rewards/incentives. | | | | | Low value; certain | | Difference in change in minutes of moderate-vigorous physical activity (mins/week) at 6 months.  Validation: minutes of physical activity measured via Global Physical Activity Questionnaire | | Assessed.  Incentive scheme duration was 12 weeks and last assessment was at 6 months | | | **Data provided by author** | | |  |
| Jeffery 1998 | | | See Jeffery 1998 under *Indicators of healthier eating and/or physical activity* | | | | | | | | | | | | | | | | | | | | | |  |
| Wing 1996  RCT | | | USA; community | | | 37 participants were recruited by newspaper ads | | Not known | | 2 groups:  **1. Control:** Participants attended a standard behavioral weigh loss prog: meetings over 24 wks w/ weigh-ins+ educational lectures+supervised walk sessions  **2. Monetary incentive:** As above but also participants earned prizes for exercise attendance ($50 gift certificate drawing after attending each walk and $2000 travel certificate drawing after last session) +participated in wkly group meetings for 24 wks w/ weigh-ins weighed +educational lectures. | | | | | High value; uncertain | | Difference in % of exercise sessions attended by incentive vs. control groups; Proportion of exercise sessions attended and proportion of participants with good attendance at 24 weeks | | Not assessed. Incentives removed at final assessment (6 months). | | | Paper reports two studies. Study 1 is excluded (does not involve incentives) Study 2 is included | | |  |

**Table S2. Results of included studies**

| **Study** | **Denominator** | **Timing of last assessment** | **Outcome** | **Results** | **Statistical significance** | **Other results** | **Comment** |
| --- | --- | --- | --- | --- | --- | --- | --- |
| ***Smoking cessation*** | | | | | | | |
| Crowley 1995 | I=18  C=15 | 6 months from intervention start  (>3-6 months after incentive removal) | Mean CCO levels | I=20.4 (SEM=4.7)  C=22.4 (SEM=4.2) | NS | Quite rate: n=5/36 | CSR group (n=16) excluded from analyses with mean CCO 23.9 (+ 3.0), Groups collapsed at follow-up when reporting quite rate |
| Donatelle 2000a | I=103  C=102 | >6-12 months from intervention start | Quit rate | I=21.4%  C=5.9% | S |  |  |
| Donatelle 2000b | I=67  C=60 | 6 months from intervention start | Quit rate | I=19.4%  C=11.7% | Not reported |  | Best Practice 5 A’s plus $25/month voucher plus biochemical feedback of potential harm to baby (n=59) group excluded from analyses with reported quire rate 22% |
| Donatelle 2002 | I(A)=192  I(B)=186  C=188 | >12-18 months from intervention start  (>6 months after incentive removal) | Quit rate | I(A)=5%  I(B)= 7%  C=3% | Not reported |  | Unpublished study. Data provided by author |
| Gallagher 2007 | I=60  C=60 | >6-12 months from intervention start | Quit rate | I=7%  C=5% | NS |  | CR+NRT (n=60) group not included in analyses with quit rate 2% |
| Gine, 2010 | I=781  C=616 | >6-12 months from intervention start  (>3-6 months after incentive removal) | Quit rate | I=11%  C=7% | S |  | Cue cards group (n=603) excluded from analyses with quit rate 2/603 |
| Glasgow 1993 | I=474  C=623 | >18 months from intervention start  (>6 months after incentive removal) | Quit rate | I=10%  C=8% | NS | Incentives had a sig. effect on less educated participants (18.6% vs. 8.8%) |  |
| Gomel 1993 | I=30  C=30 | >6-12 months from intervention start  (>3-6 months after incentive removal) | Quit rate | I=3%  C=10% | NS | Other outcomes: mean BMI change (sig. greater in HRA and RFE groups than for BC and BCI groups) mean change in % body fat (ns differences); mean change in aerobic capacity (ns differences); mean change in cholesterol (ns differences) | The health risk assessment (n=40) and risk factor education (n=28) groups were excluded. Quite rates were 5% and 6% respectively. |
| Heil 2008 | I=37  C=40 | >12-18 months from intervention start  (>2-3 months after incentive removal) | Quit rate | I=8%  C=3% | NS |  |  |
| Hennrikus 2002 | I=1264  C=1138 | >18 months from intervention start  (>6 months after incentive removal) | Quit rate | I=19.5%  C= 19.7% | Not reported |  | Groups with different mode of delivery (telephone vs group session) were combined for the analyses |
| Higgins 2004 | I=30  C=23 | >12-18 months from intervention start  (>2-3 months after incentive removal) | Quit rate | I=27%  C=0% | S |  |  |
| Higgins unpublished  (mentioned in Higgins 2012) | I=21  C=20 | >12-18 months from intervention start  (>2-3 months after incentive removal) | Quit rate | I=5%  C=0% | NS |  |  |
| Jason 1997 | I=281  C=280 | >18 months from intervention start  (>6 months after incentive removal) | Quit rate | I=13.2%  C=10.3 | NS |  | GIM group (n=283) with quit rate: 18.2% excluded from analyses |
| Jeffery 1990 (A) | I=9  C=133 | 6 months from intervention start | Quit rate | I=22%  C=6% | Not reported |  |  |
| Klesges 1987 | I=66  C=61 | 6 months from intervention start | Quit rate | I=12%  C=11.4% | Not reported |  | Relapse prevention vs no relapse prevention groups were collapsed for the analyses |
| Rand 1989 | I=16  C=16 | 6 months from intervention start | Quit rate | I=6%  C=6% | NS |  | Control group (n=14) with quit rate 0% excluded from analyses |
| Shoptaw 2002 | I(A)=43  C(A)=43  I(B)=47  C(B)=42 | >6-12 months from intervention start  (>6 months after incentive removal) | Quit rate | I(A)=2.3%  C(A)=9%  I(B)=2%  C(B)=4.8% | NS |  | Data provided by author |
| Tevyaw 2009 | I=55  C=55 | 6 months from intervention start  (>3-6 months after incentive removal) | Quit rate | I=2%  C=5.5% | NS |  | Data extracted from Cahill & Perera (2011).  Two CM groups and to NR groups collapsed for analyses |
| Volpp 2006 | I=92  C=87 | 6 months from intervention start  (>3-6 months after incentive removal) | Quit rate | I=6.5%  C=4.6% | NS |  |  |
| Volpp 2009 | I=436  C=442 | >6-12 months from intervention start  (>3-6 months after incentive removal) | Quit rate | I=9.4%  C=3.6% | S | Incentivized participants had sig. higher rates of enrolment in smoking cessation course as well as completion rates |  |
| Windsor 1988 | I(A)=94  C(A)=95  I(B)=94  C(B)=94 | >6-12 months from intervention start  (>3-6 months after incentive removal) | Quit rate | I(A)=5.3%  C(A)=6.3%  I(B)=9.6%  C(B)=18% | Not reported |  |  |
| **Indicators of healthier eating and/physical activity** | | | | | | | |
| Bloch 2006 | I=56  C=55 | 6 months from intervention start | Achievement of at least 15% reduction in LDL-C | I=37.5%  C=14.5% | Not reported | LDL-C was reduced 17.9mg/dl (11%) in incentivized group and 5.5mg/dL (4) in control and this difference was significant | Dichotomous data extracted only and used in analyses. One intervention group excluded (nurse educator, n=60) which achieved a reduction of 17.9mg/dl (11%). 21 participants reached to goal of reducing LDL-C by at least 15% |
| Francisco 1994 | I=13  C=18 | 6 months from intervention start | Mean change in serum cholesterol level | I=0.83mg/dL (1.21-21 mg/dL)  C=0.68mg/dL (0.26-10 mg/dL) | S | The incentivized group showed 13.2% reduction in serum cholesterol levels; the control exhibited 11.3% reduction. Difference was significant. | Data not included in analyses. |
| Galbo 2011 | I=51  C=48 | >6-12 months from intervention start | Number of participants losing at least 4lb | I=23  C=14 | Not reported | Mean weight loss. I=-7.3 (sd=11.1); C=-2.1 (sd=8.3). Difference significant | Dichotomous data extracted only and used in analyses. |
| Jeffery 1983 | I(a)=16  I(b)=15  I(c )=14 | >18 months from intervention start  (>6-12 months after incentive removal) | Mean weight loss | I(a)=-8.3 (sd=8.3)  I(b)=-8.5 (sd=8.3)  I(c )=-7.3 | NS | Group conditions also included (n=17; n=14; n=13;) with mean weight changes -13.6; -15.8; -14.2 respectively | Data not included in analyses |
| Jeffery 1984 | I=73  C=40 | >6-12 months from intervention start  (>6 months after incentive removal) | Mean weight loss | I=-6.43 (sd=7.73)  C=-4.75 (sd=7.12) | Not reported |  | The two financial incentive groups (fixed amount vs increments) were collapsed for the analyses. |
| Jeffery 1990 (B) | I=105  C=106 | 6 months from intervention start | Mean weight loss | I=-9.49 (SEM=1.21)  C=-4.56(SEM=1.43) | Not reported |  |  |
| Jeffery 1993 | I (A)=41  C(A)=40  I (B)=41  C(B)=40 | >18 months from intervention start  (>6 months after incentive removal) | Mean weight loss | I(A)=-1.75 (sd=6.41)  C(A)= -3.2 (sd=6.82)  I(B)= -1.75(sd=6.41)  C(B)=-2.5 (sd=6.82) | NS |  |  |
| Jeffery 1998 | I(A)=37  C(A)=41  I(B)=36  C(B)=42 | >12-18 months from intervention start | Mean weight loss | I(A)=-4.5 (SEM=1.2)  C(A)=-3.8 (SEM=1.3)  I(B)=-5.1(SEM=1.3)  C(B)=-2.9 (SEM=1.1) | S |  |  |
| John 2011 | I=44  C=22 | >12-18 months from intervention start  (>6 months after incentive removal) | Proportion achieving 5% body weight weight loss | I=11.3%  C=9% | Not reported | Mean weight loss; NS | Two incentive groups collapsed for analyses.  Data provided by author |
| Klem 1988 | I=19  C=19 | >6-12 months from intervention start  (>2-3 months after incentive removal) | Mean weight loss | I=-4.2 (sd=5.11)  C=-0.58 (sd=1.41) | S |  | Completion group excluded from analyses. Mean weight loss for group: -0.12, sd = 5.17 |
| Kramer 1986 | I=28  C=28 | >6-12 months from intervention start | Proportion maintaining their post-treatment weight | I=32%  C=17.8% | NS | Mean weight change:  I= -17.7 (sd=18.0)  C=- 18.8 (sd=15.5) | The skills training group (n=29) was excluded from analyses with 14% participants maintaining weight loss |
| Long 2012 | I=40  C=39 | 6 months from intervention start | Mean change in HbA1c levels | I=-0.46 (CI95% -1.02-0.10)  C= -0.01 (CI 95% -0.52-0.51) | NS |  | Peer mentor group (n=39) excluded from analyses. Mean change in HbA1c: -1.07% (95%CI, 1.84%-0.31%) |
| Mahoney 1974 | I(a)=13  I(b)=11  C=14 | >6-12 months from intervention start  (>6 after incentive removal) | Reduction quotient; Number of lbs. lost | Not reported | NS |  | Data not included in analyses |
| Norton 1980 | I=13  C=8 | >6-12 months from intervention start  (>6 after incentive removal) | Weight loss; Exercise and eating behavior change | Not reported | NS |  | Data not included in analyses |
| Saccone 1978 | I(A)=16  C(A)=6  I(B)=14  C(B)=8 | >6-12 months from intervention start  (>6 after incentive removal) | Mean weight loss | I(A)=-2.38 (sd=6.48)  C(A)=-1.13 (sd=6.23)  I(B)=-4.17 (sd=7.10)  C(B)=-0.59 (ds=6.08) | Not reported |  | The no treatment control group was excluded from the analyses. Mean weight loss :+4 (sd=6.5).Reinforcements by therapist and reinforcement by sig. other groups were collapsed. The two program only control groups were also collapsed. |
| Volpp 2008 | I(A)= 19  I(B)=19  C=19 | >6-12 months from intervention start  (>2-3 months after incentive removal) | Proportion achieving 5% body weight weight loss | I(A)= 5.3%  I(B)= 5.3%  C=5.3% | Not reported | Mean weight loss;  I(A)= -6.2 (CI95% −11.67-  −0.81)  I(B)= -9.2 (CI95% −15.89- −2.47)  C=-4.40 (CI95% −9.19-  0.29)  Differences NS | Data provided by author |
| Wing 1981 | I(A)=18 I(B)=20 | >6-12 months from intervention start  (>6 after incentive removal) | Weight loss | I(A)=-20.30  I(B)=-10.95 | NS |  | Data not included in analyses |
| **Physical activity** | | | | | | | |
| Hunter 2011 | I=199  C=207 | 6 months from intervention start  (>2-3 months after incentive removal) | Mean change in number of minutes of vigorous physical activity | I=3.04 (sd=298.15)  C=-37.78 (sd=409.93) | NS |  | Data provided by author |
| Jeffery 1998 | I(A)=37  C(A)=41  I(B)=36  C(B)=42 | >12-18 months from intervention start | Mean change in number of calories spent through exercise (kcal/week) | I(A)=658(SEM=180)  C(A)=338  (SEM=179)  I(B)=664 (SEM=182)  C(B)=595 (SEM=164) | NS | Number of walk sessions attended. I(A)=65.8 (SEM=8.8)  C(A)=35 (SEM=8.4)  I(B)=103.9 (SEM=9)  C(B)=80.3 (SEM=8.3); significant differences |  |
| Wing 1996 | I=21  C=16 | 6 months from intervention start | Proportion with ‘good’ adherence to exercise sessions | I=71.4%  C=56% | NS | Attendance to exercise sessions. I=60.7% of sessions  C=52.2% of sessions. Differences NS |  |

**Table S3 Multivariable analyses**

|  | **6 months from start** | | **>6-12 months from start** | | **>2-3 months from removal** | | **>6 months from removal** | |
| --- | --- | --- | --- | --- | --- | --- | --- | --- |
|  | OR (95% CI) | P-values | OR (95% CI) | P-values | OR (95% CI) | P-values | OR (95% CI) | P-values |
| **Constant** | 1.73(0.88 to 3.40) | 0.07 | 1.26(0.40 to 4.01) |  | - | - | 0.86(0.29 to 2.55) | 0.36 |
| **Behavior** |  |  |  |  |  |  |  |  |
| Smoking cessation  **vs.**  Indicators healthier eating/PA  Smoking cessation  **vs.**  Physical activity  **Attainment certainty**  Certain  **vs.**  Uncertain  Certain  **vs.**  Certain and uncertain  **Monetary value**  High  **vs.**  Low  **Level of deprivation**  Other  **vs.**  High  **Procedure standardization bias**  Low  **vs.**  High  **Outcome measure reliability bias**  Low  **vs.**  High  Low  **Vs.**  Unclear | 0.97(0.49 to 1.93)  0.68(0.26 to 1.73)  0.55(0.11 to 2.81)  0.58(0.09 to 3.77)  0.77(0.46 to 1.29)  1.25(1.62 to 2.52)  1.10(0.55 to 2.21)  1.33(0.63 to 2.84)  0.81(0.36 to 2.13) | 0.93  0.39  0.46  0.55  0.31  0.51  0.78  0.44  0.76 | 1.12(0.38 to 3.28)  -  0.64(0.10 to 3.97)  0.58(0.11 to 2.93)  0.89(0.35 to 2.26)  2.31(0.68 to 7.85)  1.05(0.47 to 2.35)  1.15(0.22 to 6.03)  0.65(0.16 to 2.59) | 0.83  -  0.61  0.48  0.80  0.17  0.90  0.86  0.52 | -  -  -  -  -  -  -  -  - | -  -  -  -  -  -  -  -  - | 1.04(0.41 to 2.62)  -  1.15(0.38 to 3.50)  1.07 (0.14 to 8.42)  1.48(0.61 to 3.56)  0.39(0.02 to 6.49)  1.39(0.21 to 9.25)  -  4.85(0.31 to 75.52) | 0.92  -  0.77  0.94  0.31  0.43  0.67  -  0.20 |

Note: n denotes number of comparisons. Data relating to >2-3 months after incentive removal were subjected only to univariable meta-regression due to the small the number of comparison
